# Targeting the NPY/NPY1R signaling axis in mutant p53–dependent pancreatic cancer impairs metastasis

**DOI:** 10.1126/sciadv.adq4416

**Published:** 2025-03-12

**Authors:** Cecilia R. Chambers, Supitchaya Watakul, Peter Schofield, Anna E. Howell, Jessie Zhu, Alice M. H. Tran, Nadia Kuepper, Daniel A. Reed, Kendelle J. Murphy, Lily M. Channon, Brooke A. Pereira, Victoria M. Tyma, Victoria Lee, Michael Trpceski, Jake Henry, Pauline Melenec, Lea Abdulkhalek, Max Nobis, Xanthe L. Metcalf, Shona Ritchie, Antonia Cadell, Janett Stoehr, Astrid Magenau, Diego Chacon-Fajardo, Jessica L. Chitty, Savannah O’Connell, Anaiis Zaratzian, Michael Tayao, Andrew Da Silva, Ruth J. Lyons, Leonard D. Goldstein, Ashleigh Dale, Alexander Rookyard, Angela Connolly, Ben Crossett, Yen T. H. Tran, Peter Kaltzis, Claire Vennin, Marija Dinevska, Australian Pancreatic Cancer Genome Initiative (APGI), Australian Pancreatic Cancer Matrix Atlas (APMA), David R. Croucher, Jaswinder Samra, Anubhav Mittal, Robert J. Weatheritt, Andrew Philp, Gonzalo Del Monte-Nieto, Lei Zhang, Ronaldo F. Enriquez, Thomas R. Cox, Yan-Chuan C. Shi, Mark Pinese, Nicola Waddell, Hao-Wen Sim, Tatyana Chtanova, Yingxiao Wang, Anthony M. Joshua, Lorraine Chantrill, Thomas R. Jeffry Evans, Anthony J. Gill, Jennifer P. Morton, Marina Pajic, Daniel Christ, Herbert Herzog, Paul Timpson, David Herrmann

**Affiliations:** ^1^ Cancer Ecosystems Program, Garvan Institute of Medical Research and The Kinghorn Cancer Centre, Darlinghurst, Sydney, New South Wales, Australia.; ^2^ School of Clinical Medicine, Faculty of Medicine and Health, University of New South Wales (UNSW), Kensington, Sydney, New South Wales, Australia.; ^3^ Immune Biotherapies Program, Garvan Institute of Medical Research, Darlinghurst, Sydney, New South Wales, Australia.; ^4^ VIB-KU Leuven Center for Cancer Biology, Leuven, Belgium.; ^5^ Translational Oncology Program, Garvan Institute of Medical Research and The Kinghorn Cancer Centre, Darlinghurst, Sydney, New South Wales, Australia.; ^6^ Data Science Platform, Garvan Institute of Medical Research, Darlinghurst, Sydney, New South Wales, Australia.; ^7^ Sydney Mass Spectrometry, University of Sydney, Sydney, New South Wales, Australia.; ^8^ Australian Regenerative Medicine Institute, Monash University, Clayton, Victoria 3800, Australia.; ^9^ Department of Microbiology and Immunology, University of Melbourne, Melbourne, Australia.; ^10^ Department of Surgery, University of Melbourne, Melbourne, Australia.; ^11^ Royal North Shore Hospital, St Leonards, Sydney, New South Wales, Australia.; ^12^ Centre for Healthy Ageing, Centenary Institute, Sydney, New South Wales, Australia.; ^13^ School of Sport, Exercise and Rehabilitation Sciences, University of Technology Sydney, Sydney, New South Wales, Australia.; ^14^ St. Vincent’s Centre for Applied Medical Research, Darlinghurst, Sydney, New South Wales, Australia.; ^15^ Children’s Cancer Institute, Lowy Cancer Research Centre, University of New South Wales (UNSW), Kensington, Sydney, New South Wales, Australia.; ^16^ QIMR Berghofer Medical Research Institute, Herston, Queensland, Australia.; ^17^ NHMRC Clinical Trials Centre, University of Sydney, Sydney, New South Wales, Australia.; ^18^ School of Biotechnology and Biomolecular Sciences, Faculty of Science, University of New South Wales, Sydney, New South Wales, Australia.; ^19^ Department of Bioengineering & Institute of Engineering in Medicine, University of California, San Diego, La Jolla, CA 92093, USA.; ^20^ Alfred E. Mann Department of Biomedical Engineering, University of Southern California, Los Angeles, CA 90089, USA.; ^21^ Department of Medical Oncology and Illawarra Shoalhaven Local Health District, Wollongong, New South Wales, Australia.; ^22^ Cancer Research UK Scotland Institute, Glasgow, UK.; ^23^ School of Cancer Sciences, Wolfson Wohl Cancer Research Centre, University of Glasgow, Glasgow, UK.; ^24^ Sydney Medical School, University of Sydney, Sydney, New South Wales, Australia.

## Abstract

Pancreatic cancer (PC) is a highly metastatic malignancy. More than 80% of patients with PC present with advanced-stage disease, preventing potentially curative surgery. The neuropeptide Y (NPY) system, best known for its role in controlling energy homeostasis, has also been shown to promote tumorigenesis in a range of cancer types, but its role in PC has yet to be explored. We show that expression of NPY and *NPY1R* are up-regulated in mouse PC models and human patients with PC. Moreover, using the genetically engineered, autochthonous KP^R172H^C mouse model of PC, we demonstrate that pancreas-specific and whole-body knockout of *Npy1r* significantly decreases metastasis to the liver. We identify that treatment with the NPY1R antagonist BIBO3304 significantly reduces KP^R172H^C migratory capacity on cell-derived matrices. Pharmacological NPY1R inhibition in an intrasplenic model of PC metastasis recapitulated the results of our genetic studies, with BIBO3304 significantly decreasing liver metastasis. Together, our results reveal that NPY/NPY1R signaling is a previously unidentified antimetastatic target in PC.

## Teaser

Targeting NPY1R reduces mutant p53–dependent pancreatic cancer metastasis.

## INTRODUCTION

Pancreatic cancer (PC) remains one of the most lethal forms of human cancer worldwide, with an estimated 5-year survival rate of only 13% (*1*). PC exhibits widespread invasion in combination with early-stage metastatic events, which leads to >45% of patients with PC presenting with distant metastatic disease at diagnosis and precludes surgical resection for most patients (~80%) (*1*, *2*). The 5-year survival rate of PC declines markedly from 44% for localized disease to 3% for distant disease (*1*), which underscores the critical need to understand which signaling pathways regulate PC metastatic colonization and to identify new ways to target these mechanisms to improve outcomes for patients with metastatic disease (*3*, *4*). While advances have been made in the treatment of PC in recent years with the addition of nab-paclitaxel (Abraxane) to gemcitabine chemotherapy (8.5-month median survival for gemcitabine/Abraxane versus 6.8 months for gemcitabine alone) and FOLFIRINOX chemotherapy (11.1-month median survival), almost all patients eventually relapse with metastatic disease and, therefore, better therapeutic options are urgently required in the clinical management of this aggressive cancer (*5*, *6*).

Pancreatic ductal adenocarcinoma (PDAC) is the most common form of PC, representing >90% of human patients. Most PDAC cases present with an activating mutation in *KRAS* (>90% of cases), resulting in the formation of precursor lesions known as pancreatic intraepithelial neoplasms (*7*–*9*). Progression to advanced stages of disease is typically accompanied by alterations in tumor suppressor genes, the most common being p53 (*TRP53*; 50 to 75% of patients with PC) (*10*, *11*). Metastasis is the result of a complex interplay between both cancer cell–autonomous functions and feedback from the tumor microenvironment (*12*–*15*). Cancer cell–autonomous metastatic processes can be driven by the genetic landscape of PC, exemplified by the gain-of-function mutation p53^R172H^, which promotes significantly increased metastasis relative to a genetic loss of p53 in *Kras^G12D^* mutant mice (*11*, *16*). Moreover, alterations in signaling pathways that regulate epithelial-to-mesenchymal transition and components that promote cancer stemness also influence the cancer cell phenotype, leading to increased metastasis (*17*, *18*). Last, pancreatic tumors are characterized by a poorly vascularized (*19*–*22*), highly fibrotic (*12*, *19*, *23*–*25*), hypoxic (*26*–*29*), and immunosuppressive tumor microenvironment (*30*), where interaction with both cellular and noncellular elements can stimulate the acquisition of invasive and motile properties within PC cells (*31*–*35*). This can lead to early and pervasive cancer cell dissemination (*36*). As metastasis is a key factor in PC-related mortality, therapies that target the cell-intrinsic and cell-extrinsic pathways that lead to metastatic spread would be of great therapeutic value.

Neuropeptide Y (NPY), together with peptide YY (PYY) and pancreatic polypeptide (PPY), form the NPY family of peptides, which in humans signals through four different G protein–coupled receptors (NPY1R, NPY2R, NPY4R, and NPY5R) (*37*, *38*). NPY is the most common neuropeptide in the central and peripheral nervous system where it regulates appetite and energy homeostasis, while PYY and PPY are gut-derived peptides acting in an endocrine and paracrine fashion to control satiety and endocrine functions (*39*, *40*). NPY signaling has also been implicated in numerous biological processes that are commonly deregulated over the course of cancer progression, including cell proliferation (*41*, *42*), immune cell function (*43*, *44*), fibrosis (*45*), neural invasion (*46*), and angiogenesis (*47*, *48*). NPY family members have been shown to increase the motility and chemotaxis of breast and prostate cancer cells in vitro (*49*, *50*) and have also been associated with metastatic spread of sarcoma in vivo (*51*), two properties that are also characteristic of PC tumorigenesis. There is also emerging evidence suggesting that NPY contributes to tumorigenesis in several different cancer types including neuroblastoma (NB) (*52*), prostate (*50*, *53*), liver (*54*), colon (*55*), and breast cancer (*56*, *57*). However, its role in PC tumor development is yet to be assessed.

Here, we identify that NPY and its receptor, *Npy1r*, are up-regulated in the highly metastatic genetically engineered KP^R172H^C mouse model of PC (*11*, *16*) and demonstrate that both ligand and receptor are expressed in the primary tumor as well as liver metastases. Pancreas-specific and whole-body *Npy1r* genetic ablation in the autochthonous KP^R172H^C model led to a significant reduction in liver metastases at the study end point. Critically, using the selective NPY1R antagonist BIBO3304, we show reduced motility of KP^R172H^C cells when migrating on cell-derived matrices (CDMs). Furthermore, we recapitulated the decrease in metastasis observed upon *Npy1r* knockout in the genetically engineered KP^R172H^C mouse model using an intrasplenic model of PC metastasis, where BIBO3304 significantly decreased metastatic burden within the liver. Overall, we reveal that NPY/NPY1R signaling is a previously unidentified target in mutant p53–dependent metastasis in PC and its inhibition may represent a potential novel antimetastatic strategy in this highly aggressive and lethal cancer.

## RESULTS

### NPY expression is up-regulated in PC

**Fig. 1.**
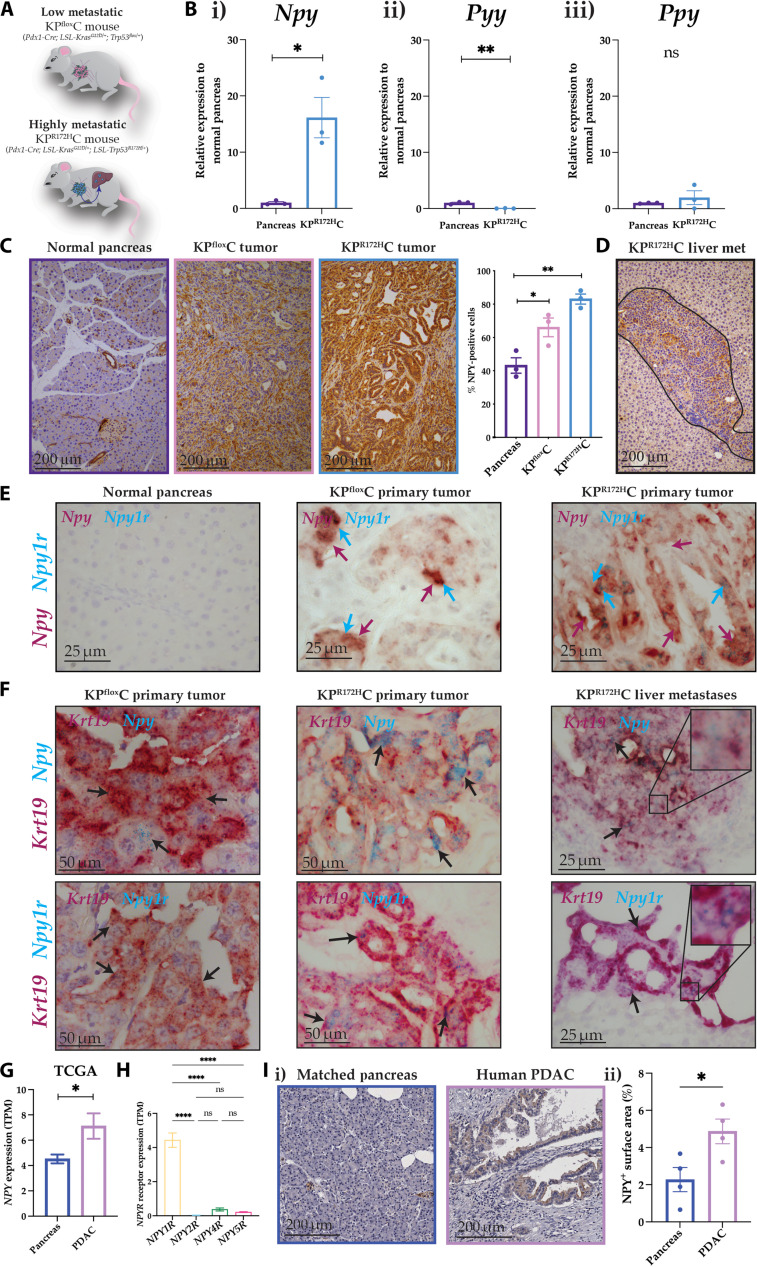
NPY expression is up-regulated in both mouse models of PC and human patients with PC. (**A**) Schematic representation of the two genetically engineered PC mouse models, KP^flox^C and KP^R172H^C (*16*). (**B**) Q-RT-PCR results of the NPY signaling ligands (i) *Npy*, (ii) *Pyy*, and (iii) *Ppy* from whole tissue samples of normal pancreas and KP^R172H^C tumors (*n* = 3). (**C**) Representative images and quantification of IHC analysis of NPY protein expression of normal pancreas and KP^flox^C and KP^R172H^C tumors (*n* = 3). Scale bars, 200 μm. (**D**) Representative image of NPY IHC of KP^R172H^C liver metastases. Scale bar, 200 μm. (**E**) Representative images of normal pancreas and KP^flox^C and KP^R172H^C tumors stained with RNAscope for *Npy* (red) and *Npy1r* (blue). Scale bars, 25 μm. (**F**) Representative images of KP^flox^C tumor and KP^R172H^C tumor with matched liver metastases stained with RNAscope for *Krt19* (red) and *Npy* (blue, top panel) or *Npy1r* (blue, bottom panel). Scale bars, 50 μm for the primary tumor and 25 μm for the liver metastases. Zoomed insets are 10 μm by 10 μm. (**G** and **H**) Gene expression data from the TCGA database assessed through OncoDB (*60*) for (G) *NPY* in human PDAC relative to normal pancreas and (H) *NPY1R*, *NPY2R*, *NPY4R*, and *NPY5R* expression from human PDAC. Normal pancreas (*n* = 200) and PDAC (*n* = 178). (**I**) (i) Representative images and (ii) quantification of IHC analysis of NPY protein expression of human PDAC tumors relative to patient matched normal pancreas (*n* = 4). Scale bars, 200 μm. Means ± SEM. ns, *P* ≥ 0.05; *P < 0.05; **P < 0.01; ****P < 0.0001 by an unpaired parametric *t* test or a one-way ANOVA with multiple comparisons.

Given the role of NPY in promoting disease progression in various other cancers, we aimed to determine whether NPY signaling could also affect pancreatic tumor development and progression. Therefore, we initially assessed the expression of NPY ligands and their receptors in two autochthonous, genetically engineered mouse models (GEMMs) of PC: the low metastatic KP^flox^C model (*Pdx1-Cre; LSL-Kras^G12D/+^; Trp53^flox/+^*; Fig. 1A) (*16*, *58*) and the highly metastatic KP^R172H^C model (*Pdx1-Cre; LSL-Kras^G12D/+^; LSL-Trp53^R172H/+^*; Fig. 1A) (*11*, *16*, *58*). In both mouse models, PC tumorigenesis is driven by a point mutation in *Kras^G12D^*, but the GEMMs are distinct in their alteration status of the tumor suppressor p53 (*Trp53*). The KP^flox^C model exhibits a loss of function of p53, while the KP^R172H^C model expresses a gain-of-function p53^R172H^ mutation, which we, and others, have previously shown drives a metastatic program to the liver relative to KP^flox^C mice (Fig. 1A) (*16*, *35*, *59*). Here, quantitative real-time polymerase chain reaction (Q-RT-PCR) analysis identified that *Npy* mRNA was significantly up-regulated in KP^R172H^C tumors relative to the normal pancreas (Fig. 1Bi), while expression of its sister peptides, peptide YY (*Pyy*) and pancreatic polypeptide (*Ppy*), was either significantly down-regulated or unchanged (Fig. 1, Bii and Biii). Next, we assessed NPY protein expression in primary PDAC tumors isolated from end-stage KP^R172H^C and KP^flox^C mice as well as in normal pancreas tissue. Immunohistochemistry (IHC) analysis revealed that NPY protein expression is significantly increased in tumors from both the KP^flox^C and KP^R172H^C GEMMs (*16*) relative to the age-matched normal pancreas (Fig. 1C). In addition, NPY is also expressed in liver metastases of the highly metastatic KP^R172H^C model (Fig. 1D), suggesting that its elevated expression is conserved during the metastatic cascade.

Moreover, this increase in NPY expression was further confirmed by chromogenic RNAscope, where staining for *Npy* (Fig. 1E, red) and its receptor *Npy1r* (Fig. 1E, blue) was readily detected in KP^flox^C and KP^R172H^C tumors relative to the normal pancreas (Fig. 1E). Next, we investigated the spatial localization of *Npy* and *Npy1r* using RNAscope by costaining with the epithelial cancer cell marker *Keratin 19* (*Krt19*). *Npy* (Fig. 1F, top panel) and *Npy1r* (Fig. 1F, bottom panel) were predominantly colocalized in cancer cells in both the KP^flox^C and KP^R172H^C primary tumors (Fig. 1F). Both *Npy* and *Npy1r* expression was retained in KP^R172H^C primary tumor and matched liver metastases, suggesting that it is maintained during metastatic spread (Fig. 1F). Overall, these data show that expression of *Npy*/NPY and its receptor *Npy1r* is up-regulated in PC mouse models compared to the normal pancreas and warrant further investigation into the role of NPY signaling through NPY1R in PC tumorigenesis.

To determine whether NPY expression was also up-regulated in tumors of human patients with PC, and to assess the clinical relevance of targeting NPY, we next interrogated the publicly available The Cancer Genomic Atlas (TCGA) dataset via OncoDB (*60*) and found that *NPY* mRNA expression was significantly elevated in PDAC tumors relative to the normal pancreas (Fig. 1G). This suggests that NPY could also play a role in tumor development in human patients with PC. Furthermore, we identified that of the four different NPY receptors (*NPY1R*, *NPY2R*, *NPY4R*, and *NPY5R*), *NPY1R* was the most highly expressed NPY receptor in human PDAC samples (Fig. 1H). Last, to confirm that NPY was also expressed at the protein level in human patients with PC, we performed IHC on human PDAC samples with the matched adjacent normal pancreas and found that NPY protein expression was significantly higher in tumors relative to the adjacent normal pancreas (Fig. 1Ii; quantified in Fig. 1Iii). Together, these results show that NPY and its receptor *NPY1R* are up-regulated in both mouse and human PC tumors, suggesting a role for NPY signaling in PC tumor development and progression.

### Conditional and whole-body knockout of *Npy1r* in the KP^R172H^C model reduces metastasis to the liver

**Fig. 2.**
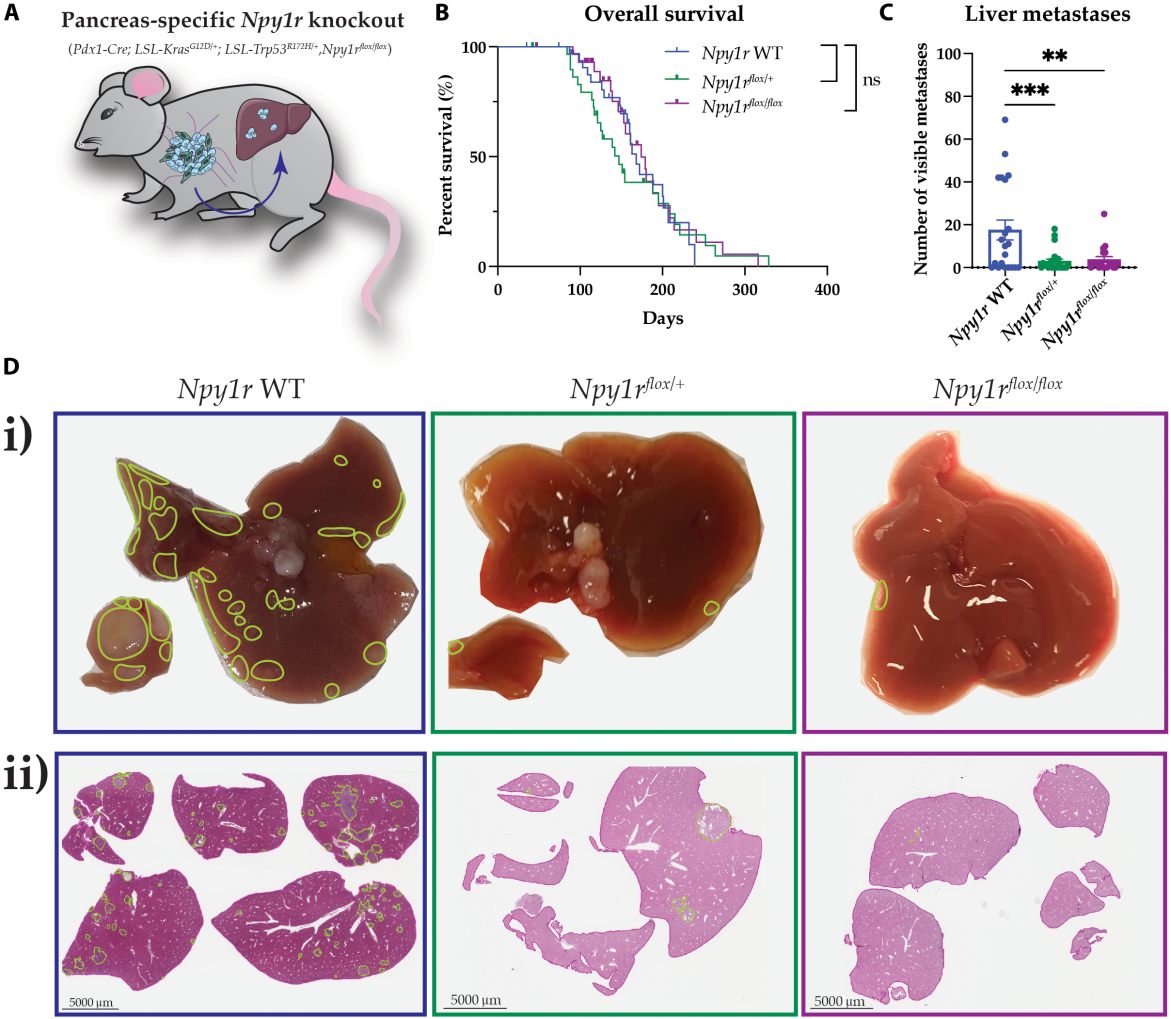
Pancreas-specific *Npy1r* genetic ablation decreases metastasis to the liver in the KP^R172H^C mouse model. (**A**) Schematic showing the pancreas-specific *Npy1r* knockout KP^R172H^C model (knockout tissue in blue). (**B**) Kaplan-Meier analysis of survival of KP^R172H^C mice for the three different pancreas-specific *Npy1r* knockout genotypes, *Npy1r* WT, *Npy1r^flox/+^*, and *Npy1r^flox/flox^* (*n* ≥ 20 mice per genotype). (**C**) Quantification of visible macrometastases in the liver at the study end point. (**D**) (i) Representative images and (ii) H&E images of sections through the livers at the study end point of the pancreas-specific *Npy1r* knockout. Metastases are outlined in green. Data for *Npy1r* WT were the same for both pancreas-specific and whole-body *Npy1r* knockout survival studies and are also shown in Fig. 3D. Scale bars, 5 mm. Means ± SEM. ns, *P* ≥ 0.05; **P < 0.01; ***P < 0.001 by a one-way ANOVA or Kaplan-Meier survival analysis.

Given that *Npy*/NPY and *Npy1r* expression is up-regulated in the highly metastatic KP^R172H^C mouse model and *NPY1R* was the most highly expressed receptor in human PDAC, we next wanted to investigate the effect of genetic ablation of *Npy1r* on disease progression in the genetically engineered KP^R172H^C mouse model of PC. To distinguish between PC cell–autonomous and nonautonomous NPY1R functions, we crossed KP^R172H^C mice with *Npy1r^flox/flox^* mice for conditional *Npy1r* knockout in the pancreatic epithelium as well as with *Npy1r^−/−^* mice to generate a whole-body *Npy1r* knockout in the KP^R172H^C model. In the pancreas-specific model, *Npy1r* knockout was driven under the *Pdx1-Cre* promoter, causing *Npy1r* to be depleted in all cells also expressing *Kras^G12D^* and *Trp53^R172H^* mutations. We generated long-term cohorts of KP^R172H^C *Npy1r* wild-type mice [*Pdx1-Cre, LSL-Kras^G12D/+^, LSL-Trp53^R172H/+^, Npy1r^+/+^* (*Npy1r* WT)], heterozygous pancreas-specific *Npy1r* knockout KP^R172H^C mice [*Pdx1-Cre, LSL-Kras^G12D/+^, LSL-Trp53^R172H/+^, Npy1r^flox/+^* (*Npy1r*^*flox/+*^)], and homozygous pancreas-specific *Npy1r* knockout KP^R172H^C mice [*Pdx1-Cre*, *LSL-Kras^G12D/+^*, *LSL-Trp53^R172H/+^*, *Npy1r^flox/flox^* (*Npy1r*^*flox/flox*^; Fig. 2A)]. Animals were closely monitored for palpable tumors and were euthanized upon reaching humane end points, as previously described (*16*). Overall, conditional *Npy1r* knockout was well tolerated with no significant change in body weight observed between the different genotypes (fig. S1A).

While Kaplan-Meier analysis of survival showed that conditional *Npy1r* knockout in the pancreas did not affect the overall survival [median survival of 168 days for *Npy1r* WT versus 147 days for *Npy1r^flox/+^* versus 174 days for *Npy1r^flox/flox^* (Fig. 2B)], we observed a significant decrease in metastasis to the liver at the study end point upon both heterozygous and homozygous pancreas-specific knockout of *Npy1r* relative to KP^R172H^C *Npy1r* WT mice (Fig. 2, C, Di, and Dii). This suggests that NPY1R signaling in PC cells may be required for metastasis to the liver in the KP^R172H^C model, which is the most common site of metastasis in human patients with PC. This observation did not occur at other PC metastatic sites, e.g., lung, diaphragm, or peritoneum, following conditional *Npy1r* ablation (fig. S1B); however, metastasis to these organs was comparatively low across all genotypes when compared with the liver, consistent with this model and clinical features in the human setting.

**Fig. 3.**
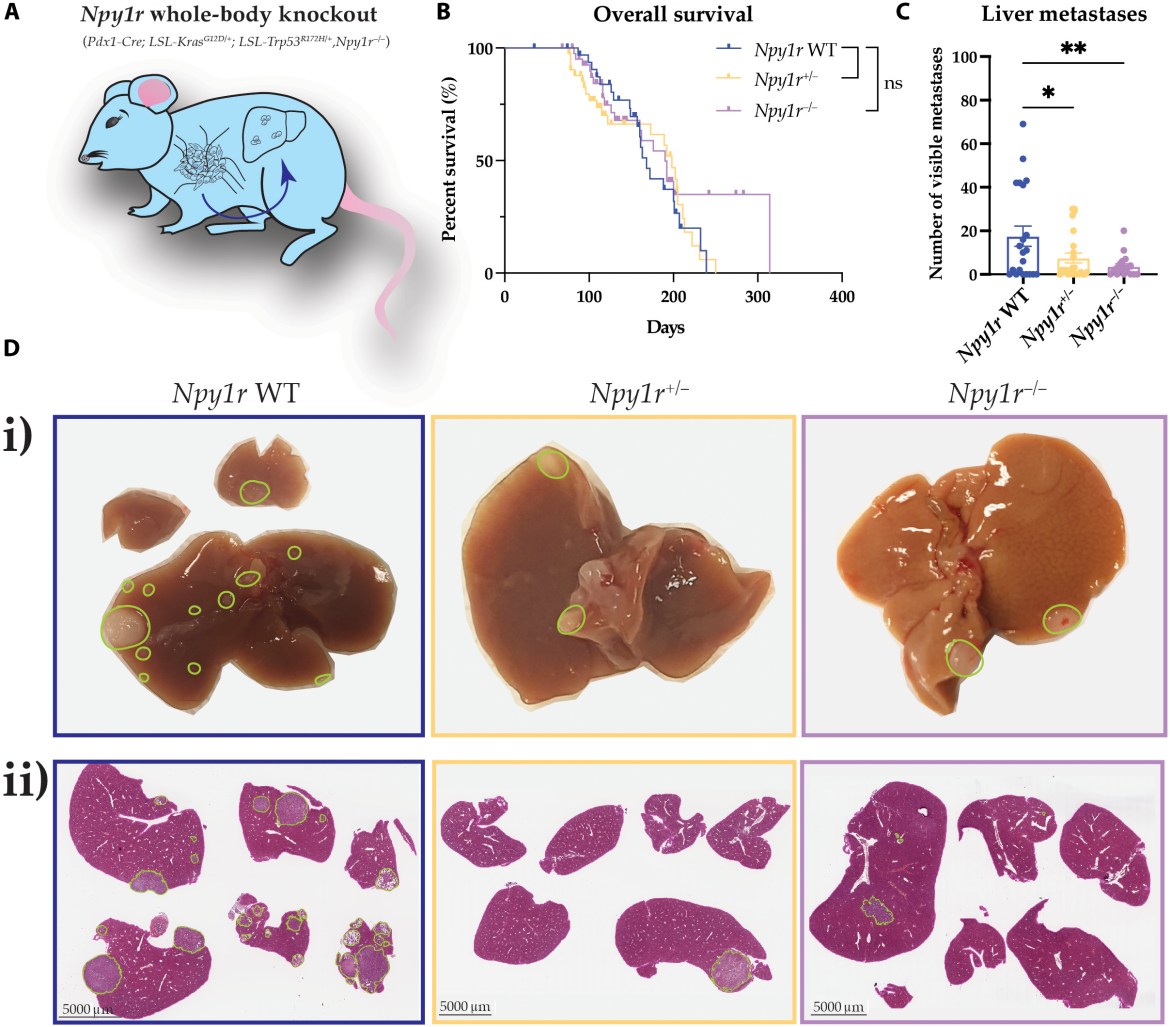
Whole-body *Npy1r* genetic ablation decreases metastasis to the liver in the KP^R172H^C mouse model. (**A**) Schematic showing the whole-body *Npy1r* knockout KP^R172H^C model (knockout tissue in blue). (**B**) Kaplan-Meier analysis of survival of KP^R172H^C mice for the three different whole-body *Npy1r* knockout genotypes, *Npy1r* WT, *Npy1r^+/−^*, and *Npy1r^−/−^* (*n* ≥ 19 mice per genotype). (**C**) Quantification of visible macrometastases in the liver at the study end point. (**D**) (i) Representative images and (ii) H&E images of sections through the livers at the study end point of the whole-body *Npy1r* knockout. Metastases are outlined in green. Data for *Npy1r* WT were the same for both pancreas-specific and whole-body *Npy1r* knockout survival studies and are also shown in Fig. 2D. Scale bars, 5 mm. Means ± SEM. ns, *P* ≥ 0.05; *P < 0.05; **P < 0.01 by a one-way ANOVA or Kaplan-Meier survival analysis.

Furthermore, to assess non–cancer cell–autonomous contributions of NPY signaling through NPY1R, we crossed the KP^R172H^C model with *Npy1r^−/−^* whole-body knockout mice. For this, we generated long-term cohorts of *Npy1r* wild-type KP^R172H^C mice [*Pdx1-Cre, LSL-Kras^G12D/+^, LSL-Trp53^R172H/+^, Npy1r^+/+^* (*Npy1r* WT)], *Npy1r* heterozygous knockout KP^R172H^C mice [*Pdx1-Cre, LSL-Kras^G12D/+^, LSL-Trp53^R172H/+^, Npy1r^+/−^* (*Npy1r*^*+/−*^)], and *Npy1r* homozygous knockout KP^R172H^C mice [*Pdx1-Cre*, *LSL-Kras^G12D/+^, LSL-Trp53^R172H/+^, Npy1r^−/−^* (*Npy1r*^*−/−*^; Fig. 3A)]. As before, all animals were closely monitored for palpable tumors and all animals were euthanized upon reaching humane end points. Here, we also observed no overall changes in body weight upon whole-body *Npy1r* knockout (fig. S1C). We found that *Npy1r* whole-body knockout in the KP^R172H^C GEMM mimicked our results from the conditional *Npy1r* knockout, exhibiting no overall changes in survival [median survival of 168 days for *Npy1r* WT versus 198 days for *Npy1r^+/−^* versus 190 days for *Npy1r^−/−^* (Fig. 3B; compared to Fig. 2B)] but again showing a significant decrease in metastasis to the liver relative to KP^R172H^C *Npy1r* WT mice (Fig. 3, C, Di, and Dii). Moreover, there were no significant changes in metastasis to organ sites outside of the liver (fig. S1D), consistent with the *Npy1r* conditional knockout model.

We also determined the time to palpable tumor and time from palpable tumor to the end stage for conditional and whole-body *Npy1r* knockout animals and found no significant difference compared to KP^R172H^C *Npy1r* WT mice (fig. S2, A and B), suggesting that the decrease in liver metastasis observed upon *Npy1r* knockout is not due to a change in overall disease progression. Moreover, because of the known role of NPY/NPY1R signaling in energy homeostasis control (*40*, *61*, *62*), we quantified the weights of key organs and adipose and muscle tissues of our mice at the end stage (fig. S3, A and B). Here, we found that while *Npy1r* knockout did not affect most organs measured, we observed significant increases in white adipose tissue weight and muscle weight, which may provide an additional benefit in PC, which is commonly associated with tissue wasting (*63*–*65*).

The pancreatic tumor microenvironment is a complex ecosystem consisting of extracellular matrix (ECM) as well as a plethora of different cell types including cancer-associated fibroblasts, endothelial cells, and immune cells, which have all previously been shown to regulate cancer metastasis to distant sites (*12*, *19*, *35*, *66*–*69*). We therefore assessed the pancreatic tumor microenvironment in end-stage tumors of the whole-body *Npy1r* knockout using IHC. This revealed that genetic *Npy1r* ablation did not significantly alter the abundance of stromal cell populations or ECM including cancer-associated fibroblasts [αSMA (ACTA2) and PDGFRB; fig. S4, A and B], endothelial cells [CD31 (PECAM1); fig. S4C], and fibrillar collagens assessed by transmitted and polarized light imaging of Picrosirius Red (fig. S5, A and B). Furthermore, no significant difference in the abundance of T cell subsets (CD4, CD8, and FOXP3; fig. S6, A to C), macrophages, or neutrophils (F4/80, ELANE, and MPO; fig. S7, A to C) was detected upon *Npy1r* knockout. These data therefore suggest that the decreased metastasis we observed upon *Npy1r* knockout was not due to changes in the overall composition of the primary tumor microenvironment.

We next assessed changes in gene expression upon *Npy1r* knockout using RNA sequencing (RNA-seq) and mass spectrometry (MS) proteomics. This revealed that 107 transcripts and 427 proteins were differentially expressed between end-stage tumors of KP^R172H^C *Npy1r* WT and KP^R172H^C *Npy1r^−/−^* mice (figs. S8, A and B, and S9, A and B, and tables S1 and S2), which may contribute to the different metastatic capacity observed for both genotypes. Down-regulated transcripts and proteins included genes previously described to be regulated by NPY (*70*), such as CCN2, whose inhibition has been shown to decrease bone metastases of MDA-MB-231 breast cancer cells (*71*), as well as CIRBP, which was previously found to be required for metastasis in a xenograft mouse model of bladder cancer (*72*). Similarly, we found significant down-regulation of LAMA3 mRNA and protein, whose expression has previously been correlated with liver metastasis in patients with PC (*73*). Up-regulated transcripts and proteins included LGALS2, which has been implicated to restrain tumor progression in colorectal and breast cancer (*74*, *75*), as well as CRABP2, whose overexpression has been associated with decreased invasiveness of ER+ breast cancer cells (*76*). Gene set enrichment analysis (GSEA) enrichment also identified a down-regulation in transforming growth factor–β signaling, coagulation, and hypoxia hallmarks (figs. S8B and S9B and tables S1 and S2), which have previously been associated with metastasis (*77*–*80*). Further studies are needed to elucidate how these alterations in gene expression programs contribute to the decrease in metastasis following *Npy1r* knockout.

Together, our GEMM studies support the notion that NPY signaling through NPY1R is required for the metastatic phenotype in KP^R172H^C cancer cells with no further reduction in metastases to the liver upon whole-body *Npy1r* knockout compared to the pancreas-specific *Npy1r* knockout in KP^R172H^C mice (Fig. 2C compared with Fig. 3C). Heterozygous *Npy1r* knockout in both the conditional and whole-body settings was sufficient to decrease metastasis to the liver, thereby suggesting that pharmacological inhibition of NPY1R function instead of complete *Npy1r* ablation may be a feasible approach to decrease PC metastasis. These results suggest that the NPY/NPY1R signaling axis could be a novel target in PC metastasis and is required for mutant p53–dependent metastatic spread to the liver in KP^R172H^C mouse models, highlighting NPY1R as a previously unidentified target in PC.

### Pharmacological NPY1R inhibition reduces the motility of KP^R172H^C cells

**Fig. 4.**
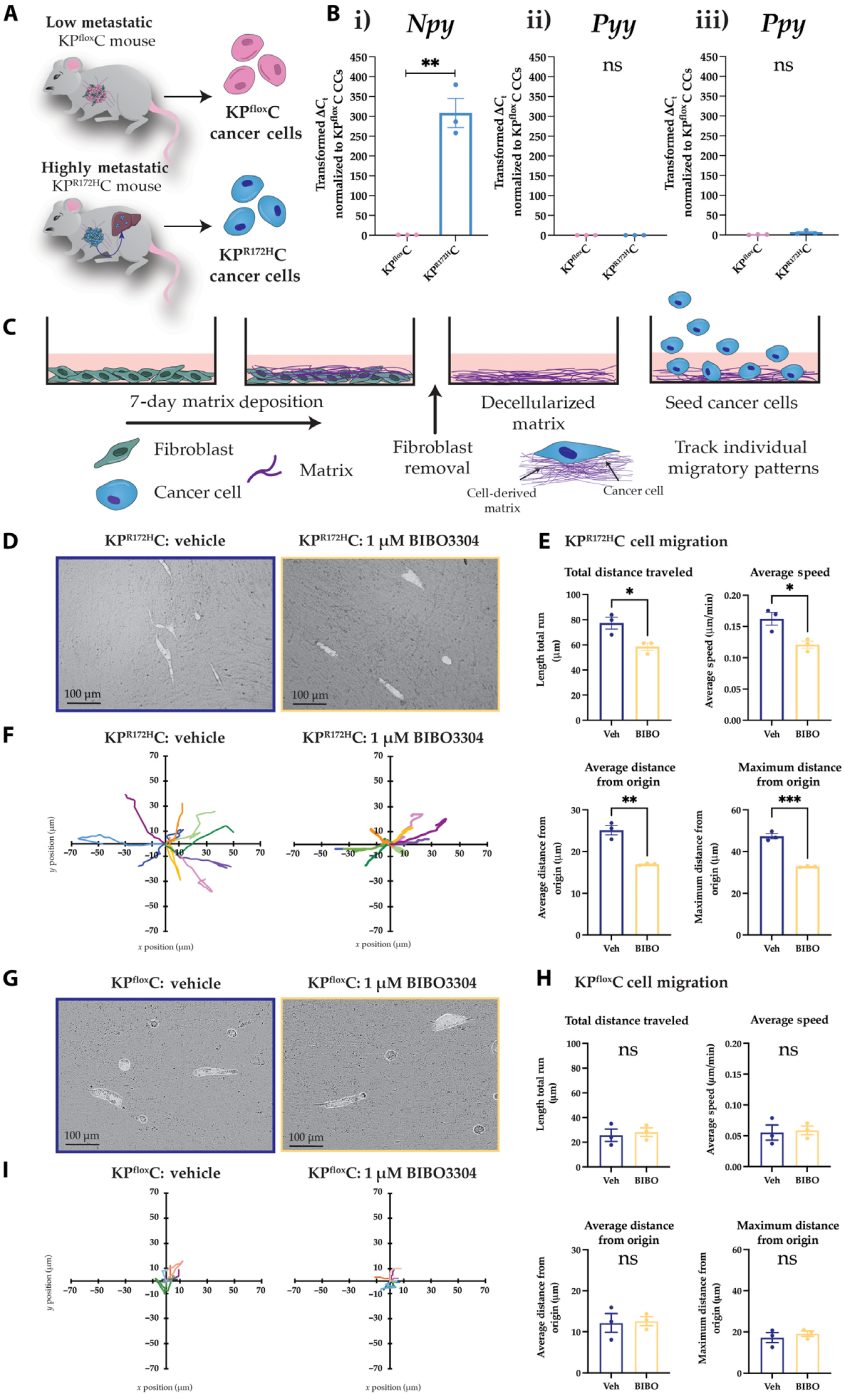
Pharmacological NPY1R inhibition decreases KP^R172H^C cell motility but not KP^flox^C motility in vitro. (**A**) Schematic showing the low metastatic KP^flox^C model and highly metastatic KP^R172H^C PC mouse models, from which cancer cells were derived and used in the following experiments. (**B**) Quantification of (i) *Npy*, (ii) *Pyy*, and (iii) *Ppy* expression using Q-RT-PCR of cancer cells isolated from the KP^flox^C and KP^R172H^C models. (**C**) Schematic showing CDM generation, decellularization, and cancer cell seeding in vitro. (**D**) Representative binary images of KP^R172H^C cells migrating on CDMs upon BIBO3304 or vehicle treatment. (**E**) Quantification of KP^R172H^C cell total distance traveled, average speed, average distance from the origin, and maximum distance from the origin treated with vehicle or 1 μM BIBO3304 over 8 hours (*n* = 3). (**F**) Representative *x*-*y* tracks of KP^R172H^C cells over 8 hours upon treatment with vehicle or 1 μM BIBO3304 (*n* = 3, 10 cell tracks per polar plot). (**G**) Representative binary images of KP^flox^C cells migrating on CDMs upon BIBO3304 or vehicle treatment. (**H**) Quantification of KP^flox^C cell total distance traveled, average speed, average distance from the origin, and maximum distance from the origin treated with vehicle or 1 μM BIBO3304 over 8 hours (*n* = 3). (**I**) Representative *x*-*y* tracks of KP^flox^C cells over 8 hours upon treatment with vehicle or 1 μM BIBO3304 (*n* = 3, 10 cell tracks per polar plot). Scale bars, 100 μm. Means ± SEM. ns, *P* ≥ 0.05; *P < 0.05; **P < 0.01; ***P < 0.001 by an unpaired parametric *t* test.

Following the observation that pancreas-specific, genetic ablation of *Npy1r* in the KP^R172H^C mouse model significantly reduced metastasis to the liver, which was not further improved upon by whole-body *Npy1r* knockout, we next aimed to elucidate how PC cell–autonomous NPY1R inhibition might be driving this antimetastatic effect. Previous studies have shown that NPY signaling stimulates cancer cell motility and invasive potential in vitro in breast and prostate cancer cell lines (*49*, *50*, *80*, *81*), warranting further investigation of the role of NPY1R in PC cell migration. We first assessed the expression of *Npy* in previously characterized primary cancer lines isolated from the low metastatic KP^flox^C mouse model compared to the highly metastatic KP^R172H^C mouse model (Fig. 4A) (*16*, *82*). To continue the assessment of NPY1R function in an immunocompetent setting, for the KP^R172H^C cell line, we used the well-characterized syngeneic TB32043 cell line, which was isolated from KP^R172H^C mice backcrossed with the C57BL/6J background (*11*, *83*, *84*). We found that *Npy* mRNA expression was significantly increased in primary cancer cells of the C57BL/6J syngeneic KP^R172H^C model relative to primary cancer cells of the KP^flox^C model via Q-RT-PCR (Fig. 4Bi), suggesting that *Npy* is important for the maintenance of the highly metastatic phenotype of the KP^R172H^C cancer cells. Notably, the up-regulation of *Npy* expression in KP^R172H^C cells compared to KP^flox^C cells (Fig. 4Bi) was enhanced compared to our immunohistochemical results in tumors (Fig. 1C), suggesting that *Npy* expression may depend on the local tumor microenvironment and/or an enhancement of *Npy* expression in KP^R172H^C cells upon backcross with the C57BL/6J background. Expression of its sister peptides *Pyy* and *Ppy* was unchanged or undetectable in KP^R172H^C cancer cells relative to KP^flox^C cancer cells (Fig. 4, Bii and Biii), further confirming that *Npy*, and not its sister peptides, may have a role in the metastatic spread in the KP^R172H^C mouse model of PC.

Next, CDMs were generated as previously described (*85*, *86*), where fibroblasts are treated with ascorbic acid to induce ECM deposition (Fig. 4C). After 7 days, the CDMs were decellularized and primary KP^R172H^C cancer cells seeded onto the CDMs to monitor their migratory patterns on a three-dimensional ECM scaffold (Fig. 4, C and D; see movie S1). Using primary KP^R172H^C cancer cells, we tracked cancer cell movement on CDMs upon pharmacological NPY1R inhibition using the NPY1R antagonist BIBO3304 (Fig. 4, C to F) (*87*) to assess how inhibiting the NPY signaling axis might affect PC cell motility. BIBO3304 is a selective small-molecule NPY1R antagonist exhibiting high affinity and specificity for NPY1R (*87*–*89*). The total distance the cancer cells traveled, the average speed, the average distance from the point of origin, and the maximum distance from the point of origin were all significantly decreased upon NPY1R inhibition using BIBO3304 (Fig. 4, E and F), confirming that NPY1R may regulate the motility of KP^R172H^C cancer cells. Moreover, BIBO3304 treatment of KP^flox^C cells migrating on CDMs did not result in a significant change in any of the parameters assessed (total difference traveled, average speed, and average/maximum distance from the point of origin; Fig. 4, G to I, and movie S2), suggesting that the low basal level of migration observed in KP^flox^C cells does not require NPY/NPY1R signaling. Together, these results reveal a decrease in mutant p53–dependent PC cell motility upon NPY1R inhibition, which could, in part, be contributing to the antimetastatic effect observed upon conditional and whole-body *Npy1r* knockout within the KP^R172H^C mouse model (Figs. 2 and 3).

### Pharmacological NPY1R inhibition reduces metastasis to the liver in vivo

**Fig. 5.**
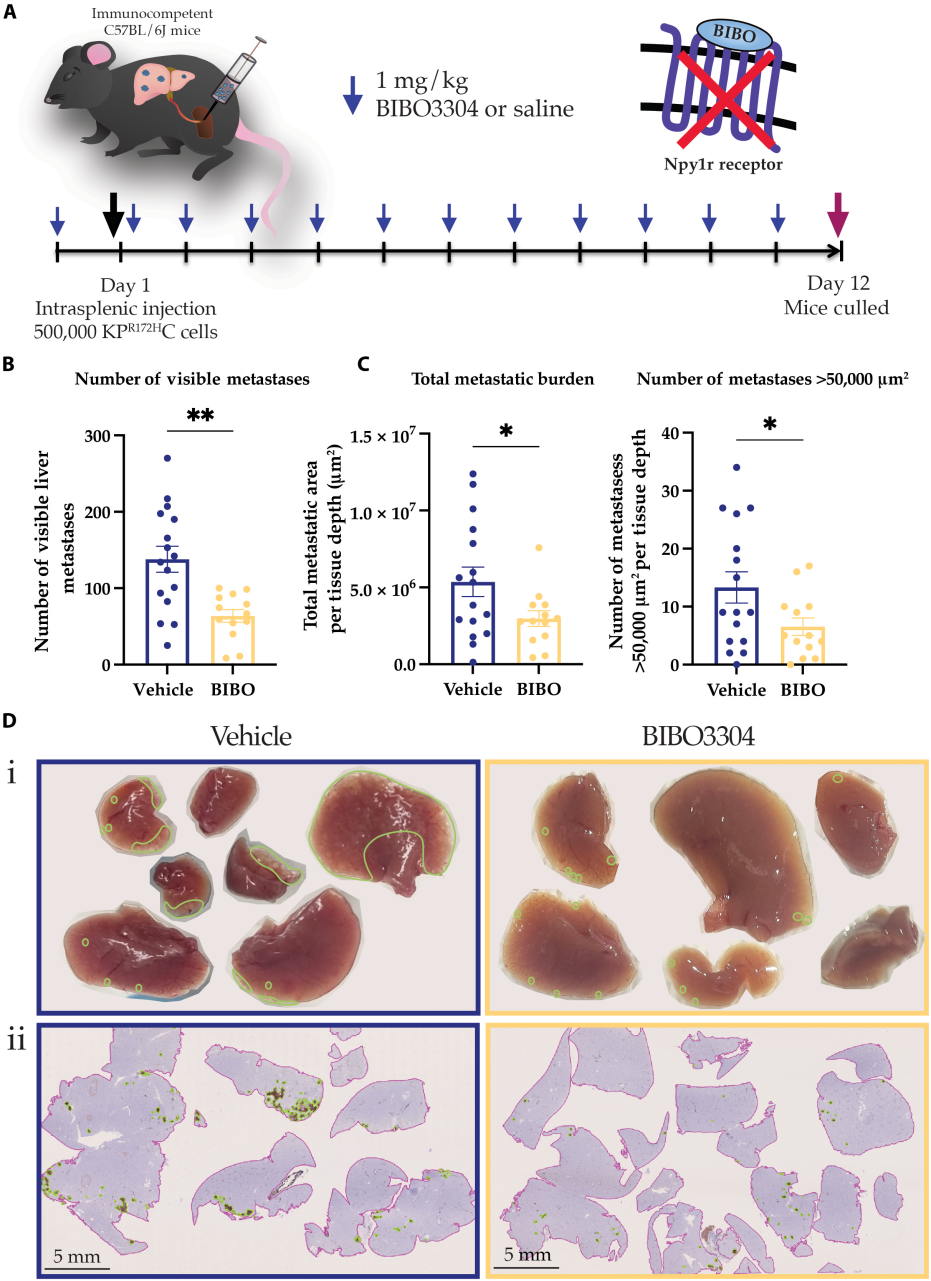
Pharmacological NPY1R inhibition decreases metastatic burden within the liver in vivo. (**A**) Schematic showing the treatment schedule for the intrasplenic xenograft experiment with BIBO3304 or vehicle control (saline). (**B**) Quantification of the number of visible liver metastases per mouse (*n* ≥ 13 mice per treatment). (**C**) Quantification of the KRT19^+^ surface area and the number of metastases >50,000 μm^2^ per tissue depth upon treatment with vehicle or BIBO3304. (**D**) (i) Representative images (top panel) and (ii) IHC images (bottom panel) of KRT19-stained livers, outlining liver tissue in pink and metastases in green, upon treatment with vehicle or BIBO3304. Scale bars, 5 mm. Means ± SEM. *P < 0.05; **P < 0.01 by an unpaired parametric *t* test.

We next sought to interrogate whether pharmacological NPY1R inhibition using BIBO3304 would recapitulate the observed decrease in liver metastasis found upon genetic *Npy1r* deletion. Therefore, we used an intrasplenic model of PC metastasis where the syngeneic KP^R172H^C cancer cells were injected into the spleens of C57BL/6J mice to drive metastasis to the liver. In this model, the flux of cancer cells through the portal vein of the spleen results in robust metastasis to the liver, as achieved previously (*33*, *90*). Mice were subjected to daily intraperitoneal injections with BIBO3304 (1 mg/kg) or vehicle before and after intrasplenic injection, resulting in pharmacological NPY1R inhibition being present during the transit and liver colonization phase of KP^R172H^C cancer cell metastasis (Fig. 5A). Mice were euthanized 12 days postsurgery (Fig. 5A). Consistent with the genetic *Npy1r* knockout models, pharmacological NPY1R inhibition resulted in significantly decreased visible metastases within the liver (Fig. 5, B and Di), further confirming that NPY1R is required for metastatic spread in PC. Livers were also stained for KRT19 to detect PC cells to further validate the antimetastatic effect of NPY1R inhibition. From this, total metastatic burden within the liver (KRT19^+^ area) and the number of large metastases (metastases >50,000 μm^2^) were assessed and were found to be significantly decreased upon BIBO3304 treatment (Fig. 5, C and Dii). These results show that pharmacological NPY1R inhibition using BIBO3304 reduces PC metastasis to the liver, highlighting the possibility that NPY1R inhibition could represent a novel future therapeutic strategy to counteract PC metastasis in combination with standard-of-care therapies, such as gemcitabine/Abraxane or FOLFIRINOX (*5*, *6*).

**Fig. 6.**
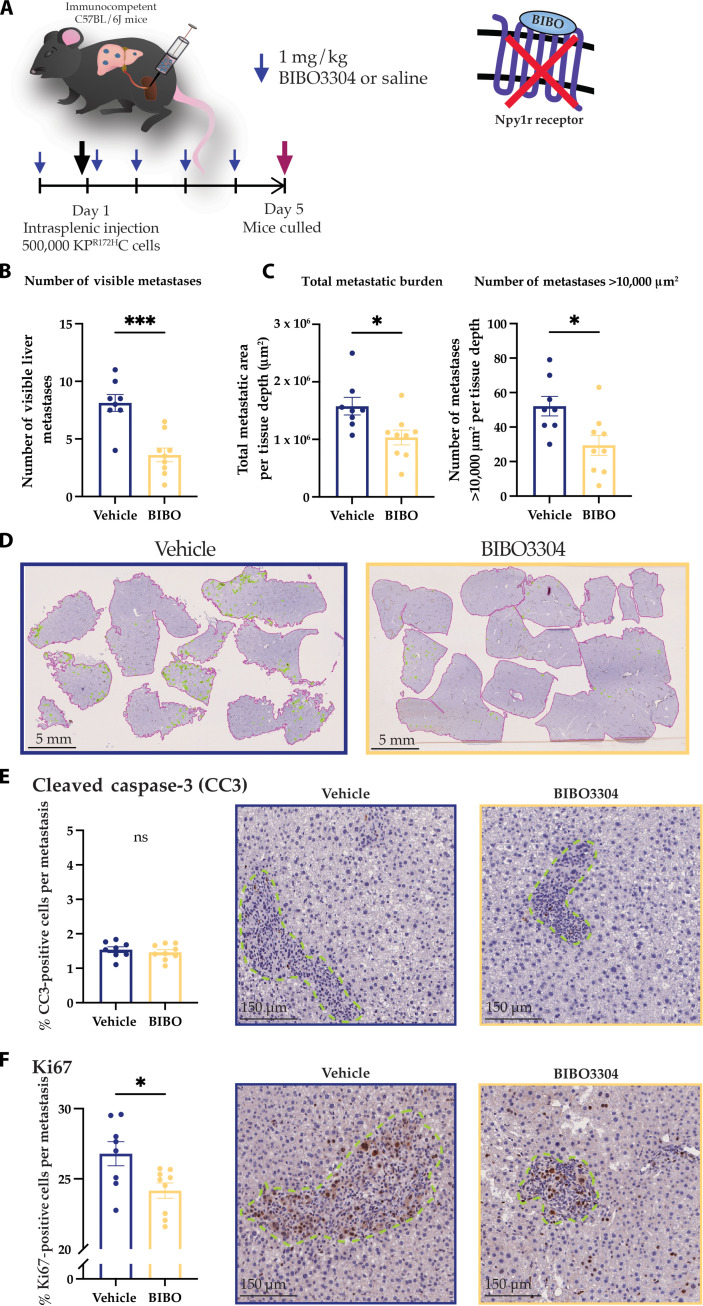
Early NPY1R inhibition decreases metastatic burden within the liver in vivo. (**A**) Schematic showing the treatment schedule for the early intrasplenic xenograft experiment with BIBO3304 or vehicle control (saline). (**B**) Quantification of the number of visible liver metastases per mouse (*n* ≥ 8 mice per treatment). (**C**) Quantification of the KRT19^+^ surface area and the number of metastases >10,000 μm^2^ per tissue depth upon treatment with vehicle or BIBO3304. (**D**) Representative IHC images of KRT19-stained livers, outlining liver tissue in pink and metastases in green, upon treatment with vehicle or BIBO3304. Scale bars, 5 mm. (**E**) Quantification and representative images of cleaved caspase-3 (CC3) IHC in liver metastases on day 5 following intrasplenic KP^R172H^C cancer cell injection and treatment with vehicle or BIBO3304 (*n* ≥ 8 mice per treatment). (**F**) Quantification and representative images of Ki67 IHC in liver metastases on day 5 following intrasplenic KP^R172H^C cancer cell injection and treatment with vehicle or BIBO3304 (*n* ≥ 8 mice per treatment). Scale bars, 150 μm. Means ± SEM. ns, *P* ≥ 0.05; *P < 0.05; ***P < 0.001 by an unpaired parametric *t* test.

To interrogate whether BIBO3304 also affects early metastatic outgrowth, we repeated the intrasplenic model and assessed metastatic burden on day 5 (4 days after intrasplenic cancer cell injection; Fig. 6A). We found that in this early intrasplenic model, BIBO3304 also significantly reduced liver metastases as assessed by both visual inspection (Fig. 6B) and KRT19 IHC (Fig. 6, C and D). Moreover, to assess whether BIBO3304 treatment affects cell survival or proliferation in these early metastases, we stained our samples for cleaved caspase-3 and Ki67. While we did not observe any changes in cell survival (Fig. 6E), we observed a significant reduction in Ki67 following BIBO3304 treatment (Fig. 6F), suggesting that BIBO3304 may reduce metastasis by decreasing cell proliferation. Collectively, these results demonstrate a requirement for NPY1R in PC metastasis and warrant further assessment of the NPY/NPY1R signaling axis as a cotarget in conjunction with current and contemporary standard-of-care therapies in PC (*5*, *6*).

## DISCUSSION

The role of the NPY signaling axis in tumorigenesis has been established in multiple cancers. For example, in NB, NPY increases tumor growth in mice xenografts (*52*), and high serum NPY correlates with decreased survival, increased metastasis, and disease recurrence in human patients with NB (*91*, *92*). In breast cancer, NPY1R expression was confirmed in 85% of primary human breast carcinomas and 100% of lymph node metastases exhibiting a switch from NPY2R expression in normal breast tissue to NPY1R dominant expression during cancer progression (*56*). In addition, high NPY1R expression in circulating tumor cells isolated from peripheral blood has also been shown to correlate with poor survival and lymph node metastasis in patients with breast cancer (*57*). Furthermore, NPY signaling was shown to regulate angiogenesis in colon cancer and tumor growth and fibrosis in liver cancer (*54*, *55*). However, NPY’s function in PC tumorigenesis has yet to be assessed. Therefore, we sought to characterize the role of NPY in PC and elucidate whether NPY is playing a role in PC tumorigenesis and progression. In this study, we found that NPY is up-regulated at both the mRNA and protein levels in the highly metastatic KP^R172H^C model of PC relative to the normal pancreas and is predominantly colocalized with cancer cells in the primary tumor and liver metastases. Furthermore, NPY expression is increased in the tumors of human patients with PDAC relative to the normal pancreas, and expression analysis confirmed that *Npy1r* is up-regulated in KP^R172H^C tumors compared with the normal pancreas. Moreover, *NPY1R* is the most highly expressed NPY receptor in human patients with PDAC, supporting a role of NPY signaling in murine and human PC tumorigenesis.

PC metastasis is a key factor in human patients’ mortality, with the 5-year survival of patients presenting with metastatic disease being only 3% (*1*). Here, we show that both heterozygous and homozygous pancreas-specific and whole-body knockout of *Npy1r* results in significantly decreased metastasis to the liver in the highly metastatic KP^R172H^C mouse model. The liver is the most common site of metastasis in human patients, with ~80% of patients with PC presenting with hepatic metastases at autopsy (*93*). Heterozygous *Npy1r* knockout in both the pancreas-specific and whole-body knockout models was sufficient to decrease metastatic burden within the liver, suggesting that complete *Npy1r* depletion is not required to impair this metastatic program. Our observations are consistent with other cancer types where NPY has been shown to play a role in metastasis (*51*, *57*, *80*). In a recent study investigating Ewing sarcoma, it was found that enhanced metastasis to the bone was driven by a hypoxia-induced activation of NPY signaling components (*51*). This observation might not be limited to Ewing sarcoma tumors, as hypoxia also induces NPY1R and NPY5R expression in breast cancer cells, causing them to migrate more upon NPY stimulation in vitro (*80*). Moreover, human patients with breast cancer whose circulating tumor cells were NPY1R positive exhibited decreased overall survival and the level of NPY1R also correlated with late-stage disease and lymph node metastases (*57*). Last, immunohistochemical analysis of human patients with prostate cancer found that NPY, NPY1R, NPY2R, and NPY5R expression was all significantly increased at the invasive border relative to the bulk tumor mass (*50*). These data, alongside our observation of decreased liver metastasis upon *Npy1r* ablation in the KP^R172H^C mouse model of PC, highlight a new role for the NPY signaling axis in PC aggressiveness.

Transcriptomic and proteomic assessment via RNA-seq and MS, respectively, identified ~500 differentially expressed transcripts and proteins in pancreatic tumors following *Npy1r* knockout. While some of these differentially expressed genes/proteins have already been shown to have a role in cancer metastasis, such as CCN2 (*71*), CIRBP (*72*), and LAMA3 (*73*), many are underexplored in PC, warranting further studies based on our datasets.

We also investigated the cancer cell–intrinsic features of NPY1R inhibition in KP^R172H^C cells and found that their three-dimensional motility was decreased upon NPY1R inhibition when migrating upon CDMs. This is consistent with studies that have found a role for NPY in regulating the motility and chemotaxis of cancer cells. For example, two breast cancer cell lines MDA-MB-231 and MCF-7 exhibit increased motility and invasion in response to NPY, which could be blocked with pharmacological NPY1R and NPY5R antagonists (*49*). Moreover, NPY was shown to regulate chemotaxis in the highly aggressive LNCaP prostate cancer cell line (*50*). The observed reduction in the motility of KP^R172H^C cells upon BIBO3304 treatment suggests a role for NPY in cancer cell movement and could represent one of the modes through which NPY1R inhibition decreases metastasis to the liver.

Our study focused on NPY1R because of its significantly increased expression in human patients with PDAC relative to NPY2R, NPY4R, and NPY5R. However, other NPY receptors have been implicated in promoting tumorigenicity in other cancers; for example, NPY2R is up-regulated in vascular endothelial growth factor A–depleted orthotopic models of colon cancer, and NPY2R antagonists inhibited angiogenesis and tumor growth in these models (*55*). Furthermore, in NB, NPY has been shown to promote cell motility via increased cytoskeleton remodeling (*94*), and NPY5R expression was significantly up-regulated in NB cells adjacent to blood vessels, suggesting preferential NPY5R expression in angioinvasive cancer cells (*92*). Therefore, other NPY receptors could be playing a role in PC and future studies could aim to elucidate their functions.

Last, we were able to recapitulate the observed decrease in liver metastases seen during *Npy1r* genetic ablation using pharmacological NPY1R inhibition in an intrasplenic model of metastasis. Here, using pharmacological NPY1R inhibition via the small-molecule antagonist BIBO3304, we show a significant decrease in metastatic burden in the liver compared with vehicle-treated mice, highlighting a potential future therapeutic strategy to reduce metastatic burden for patients. BIBO3304 exhibits subnanomolar affinity for human NPY1R (0.69 ± 0.16 nM), which is comparable to that of rodent NPY1R (0.72 ± 0.42 nM), and therefore can bind and inhibit human NPY1R (*87*) but has yet to be used in human clinical trials. The fact that BIBO3304 is unable to cross the blood-brain barrier and thus cannot affect the central action of other NPY1R-mediated functions, such as stimulating appetite and reducing energy expenditure, represents a distinct advantage in a cancer setting, since PC progression is often associated with tissue wasting (*63*–*65*). Here, we observed an increase in adipose and muscle tissue mass in *Npy1r* knockout settings, providing an additional benefit on top of reducing metastasis in PC.

Collectively, our findings establish NPY1R as a previously unidentified drug target in PC metastasis. We demonstrate that genetic and pharmacological inhibition of NPY1R reduces metastasis in the highly aggressive KP^R172H^C model. Targeting this pathway may therefore represent a highly effective novel antimetastatic strategy for future assessment in conjunction with other standard-of-care approaches, such as gemcitabine/Abraxane or FOLFIRINOX chemotherapies (*5*, *6*), to improve outcomes for patients with PC.

## MATERIALS AND METHODS

### Experimental design

This study assesses the function of NPY signaling in PC. Q-RT-PCR analysis of in vitro assays of cell lines and CDMs was conducted in independent biological triplicates with three technical replicates per repeat for each genotype or treatment group. Q-RT-PCR and IHC analysis of mouse tumor tissue and normal pancreas were performed with tumors/tissues isolated from three independent mice per genotype. IHC analysis of human PDAC tissue and matched normal pancreas was performed with tumors and tissues isolated from four patients with PC, taking four representative regions of interest (ROIs) per tissue type per patient. Human *NPY* and *NPY1R* expression was assessed via analysis of mRNA expression from the TCGA datasets through OncoDB (*60*). Mouse numbers used in in vivo experiments are outlined in the corresponding figure legends.

### Statistical analysis

Unless stated otherwise, *P* values were determined using unpaired parametric *t* test (comparison between two groups) or an unpaired one-way analysis of variance (ANOVA) assuming Gaussian distribution with Tukey’s multiple comparisons test (comparison between more than two groups). Kaplan-Meier survival curves were evaluated using a log-rank Mantel-Cox test. All statistical analysis was performed using GraphPad Prism (GraphPad Software Inc., CA) with statistical significance defined as ns, *P* ≥ 0.05; *P < 0.05; **P < 0.01; ***P < 0.001.

### Human ethics

Human ethics approval for acquisition of data and biological material was obtained from North Shore Local Health District human research ethics committee (2023/ETH02130).

### Animals

Animal experiments were conducted in compliance with the Australian code of practice for the care and use of animals for scientific purposes and the Garvan/St. Vincent’s Animal Ethics committee (guidelines 19/06, 19/10, 19/13, 22/08, 22/09, and 22/10). All experimental end points were in accordance with Garvan/St. Vincent’s Animal Ethics committee (guidelines 19/06, 19/10, 19/13, 22/08, 22/09, and 22/10). Mice were kept in ventilated cages on a 12-hour light/dark cycle with 24-hour access to food and water.

### Quantitative real-time polymerase chain reaction (Q-RT-PCR)

Q-RT-PCR on tumors from end-stage KP^R172H^C mice and normal pancreas was performed by collecting tissue samples isolated from three independent mice per genotype and immediately snap freezing the tissues before homogenization in 500 μl of RNA Prep Buffer using metal bead lysing matrix Eppendorf tubes (MP Biomedicals) in a FastPrep Tissue Homogenizer (MP Biomedicals). Q-RT-PCR on cell lines (KP^R172H^C TB32043 and KP^flox^C cancer cells) was performed by seeding three repeats of three replicates of 500,000 cells into 10-cm dishes for 48 hours. The cells were then washed twice with phosphate-buffered saline (PBS), lifted with a cell scraper, transferred to an Eppendorf tube, and spun at 13,000 rpm for 5 min at 4°C. The supernatant was removed, and the cell pellet was snap frozen and stored at −80°C. RNA extraction was performed using the RNA Qiagen Spin Column Extraction protocol (Qiagen). cDNA was synthesized using the Roche transcriptor first strand cDNA synthesis kit’s protocol (Roche). Q-RT-PCR was performed using the Universal ProbeLibrary System (UPL; Roche) on a LightCycler 480 System (Roche) using the forward and reverse primers listed in Table 1. *Gapdh* was used as control for tissues and *Rplp0* used as control for cell lines.

**Table 1.** UPL probe number and forward and reverse primer sequences for all genes assessed during Q-RT-PCR analysis.

Genes	UPL probe #	Forward	Reverse
*Npy*	96	5′-GAA AGC ACA GAA AAC GCC CCC AG-3′	5′-AAA TGG GGC GGA GTC CAG CCT A-3′
*Pyy*	17	5′-CCT ACC CTG CCA AAC CAG-3′	5′-GGA CAT CTC TTT TTC CAT ACC G-3′
*Ppy*	1	5′-TGG CTT GAT TCC CTG CTC-3′	5′-ACG GGC TGA AGA CAA GAG AG-3′
*Gapdh*	52	5′-GGG TTC CTA TAA ATA CGG ACT GC-3′	5′-CCA TTT TGT CTA CGG GAC GA-3′
*Rplp0*	9	5′-ACT GGT CTA GGA CCC GAG AAG-3′	5′-CTC CCA CCT TGT CTC CAG TC-3′

### Immunohistochemistry

Hematoxylin and eosin (H&E) and IHC staining was performed on 4-μm sections of formalin-fixed paraffin-embedded tissue with sections for H&E placed onto plain glass slides and sections for IHC placed onto positively charged slides. The slides were incubated for 2 hours in a 60°C oven for maximum adhesion. H&E sections were deparaffinized and stained following standard H&E procedures on the Leica ST5010 Autostainer XL with hematoxylin [Hematoxylin Harris nontoxic (acidified), Australian Biostain] and eosin (Eosin Phloxine Alcoholic 1%, Australian Biostain). All IHC was performed on a Leica Bond RX machine using the Bond Polymer Refine Detection Kit (DS9800), where slides were first dewaxed using Bond Dewax Solution (Leica, AR2992) before heat-induced epitope retrieval at 100°C with either epitope retrieval solution 1 (pH 6, Leica AR9961) or epitope retrieval solution 2 (pH 9, AR9640) for 20 to 40 min (please see Table 2 for details). The slides were then incubated with primary antibody for 60 min before being washed, incubated with secondary antibody (polymer), and visualized with 3,3′-diaminobenzidine. Please see Table 2 for primary antibody dilution, host source, and retrieval protocol for the antibodies used. For NPY staining of KP^R172H^C tumors, KP^flox^C tumors, and normal pancreas, tumor/tissue sections from three independent mice per genotype were stained. For human tissues, sections of tumor and adjacent normal pancreas on the same slide and isolated from five independent patients with PC were stained, and four ROIs were taken from epithelial cancer regions versus four ROIs from normal pancreas regions and averaged. For IHC analysis of KP^R172H^C *Npy1r* WT and KP^R172H^C *Npy1r^−/−^* tumors (from 10 animals with the highest number of liver metastases per genotype), 10 ROIs per tumor were analyzed. For IHC analysis of Ki67 and cleaved caspase-3 of the early intrasplenic study (day 5), 10 metastases per animal were analyzed. Slides were imaged on the Hamamatsu NanoZoomer S210 Digital slide scanner and quantified in QuPath.

**Table 2.** List of primary antibodies used for IHC with their supplier or company, catalog number, level of dilution, host source, staining system, and retrieval protocol.

Antibody	Supplier/company	Catalog no.	Antibody dilution	Host	Staining system	Retrieval
NPY	Abcam	Ab30914	1:2000	Rabbit	Leica BOND IHCF	pH 9, 20 min
KRT19	Abcam	Ab133496	1:1000	Rabbit	Leica BOND IHCF	pH 6, 40 min
Ki67	Thermo Fisher Scientific	Ab-4 Neomark	1:500	Rabbit	Leica BOND IHCF	pH 9, 30 min
CC3	Cell Signaling Technology	9661	1:200	Rabbit	Leica BOND IHCF	pH 9, 20 min
αSMA	Abcam	Ab5694	1:100	Rabbit	Envision Rabbit	pH 9, 30 min
PDGFRB	Cell Signaling Technology	3169	1:100	Rabbit	Leica BOND IHCF	pH 9, 30 min
CD31	DIA-310	Dianova	1:100	Rat	Leica BOND IHCF	pH 6, 40 min
CD4	Cell Signaling Technology	25229	1:100	Rabbit	Leica BOND IHCF	pH 9, 20 min
CD8	Cell Signaling Technology	98941	1:200	Rabbit	Leica BOND IHCF	pH 9, 20 min
FOXP3	Cell Signaling Technology	12653	1:400	Rabbit	Leica BOND IHCF	pH 9, 20 min
F4/80	Cell Signaling Technology	70076	1:100	Rabbit	Envision Rabbit	pH 9, 30 min
ELANE	Abcam	Ab68672	1:500	Rabbit	Leica BOND IHCF	pH 6, 40 min
MPO	Agilent	A039829-2	1:2000	Rabbit	Leica BOND IHCF	pH 9, 30 min

### Picrosirius Red histological staining and transmitted and polarized light imaging and analysis

Formalin-fixed paraffin-embedded tumors (from 10 animals with the highest number of liver metastases per genotype) were cut at 4-μm section thickness and allowed to adhere to Superfrost Plus slides. Slides were dewaxed on a Leica ST5010 Autostainer XL and manually stained using 0.1% Picrosirius Red and 0.02% phosphomolybdic acid for fibrillar collagens (Australian Biostain). Following a rinse in acidified water and dehydration in a graded ethanol series, slides were covered with coverslips using a Leica Coverslipper (CV5030) and imaged on a Slideview VS200 slide scanner (Olympus) in bright-field and polarized (birefringence) mode. The Picrosirius Red signal was analyzed in QuPath, and the birefringence signal was analyzed with ImageJ as described previously (*19*, *33*) with 10 ROIs per animal. To assess the red-orange, yellow, and green birefringence signals, images were analyzed using ImageJ, as described previously (*19*, *33*). Briefly, hue-saturation balance thresholding was applied (high birefringence/red-orange 0 > H < 27 | 0 > S < 255 | 70 > B < 255, medium birefringence/yellow 28 > H < 47 | 0 > S < 255 | 70 > B < 255, and low birefringence/green 48 > H < 140 | 0 > S < 255 | 70 > B < 255).

### RNAscope

RNAscope was performed using RNAscope 2.5 HD Duplex Detection kit (ADV322500), as previously described (*95*, *96*). Sections (4 μm) of formalin-fixed paraffin-embedded tissues were cut and then dehydrated, and then an ImmEdge Hydrophobic Barrier Pen (ADV310018) was used to draw a hydrophobic barrier around the tissue of interest. The tissue was then pretreated with RNAscope H_2_O_2_ & Protease Plus Reagent (ADV322330), followed by submergence in RNAscope Target Retrieval Reagent (ADV322000) and then, lastly, the addition of Protease Plus (ADV322340). RNA was then amplified and detected using probes for *Npy* (ACDBIO, 313321), *Npy1r* (ACDBIO, 427021), and *Krt19* (ACDBIO, 402941) using RNAscope 2.5 HD Duplex Reagent Kit (ADV322500). For RNAscope, a normal pancreas, KP^flox^C end-stage tumor, KP^R172H^C end-stage tumor, and the matched liver were stained. Five field-of-view images were acquired at 63× magnification on a Leica DM4000 bright-field microscope for each tissue and representative image shown.

### Expression analysis in OncoDB

The OncoDB database (https://oncodb.org/) was used to assess *NPY* expression in patients with PDAC. RNA-seq data of pancreatic tissue obtained from patients with PDAC (*n* = 178) and normal pancreas controls (*n* = 200) were analyzed for *NPY* expression [transcripts per million (TPM)] and subjected to an unpaired parametric *t* test. *NPY1R*, *NPY2R*, *NPY4R*, and *NPY5R* expression (TPM) was analyzed in patients with PDAC (*n* = 178), and an unpaired one-way ANOVA assuming Gaussian distribution with Tukey’s multiple comparisons test was performed.

### Survival studies with genetically engineered mice

KP^R172H^C mice (*Pdx1-Cre, LSL-Kras^G12D/+^, LSL-Trp53^R172H/+^*) (*11*, *16*) were crossed with *Npy1r^flox^* mice (*89*, *97*) for conditional knockout of *Npy1r* in pancreatic epithelial cells. Cohorts of KP^R172H^C *Npy1r* wild-type mice (*Pdx1-Cre, LSL-Kras^G12D/+^, LSL-Trp53^R172H/+^*, *Npy1r^+/+^*), heterozygous pancreas-specific *Npy1r* knockout KP^R172H^C mice (*Pdx1-Cre, LSL-Kras^G12D/+^, LSL-Trp53^R172H/+^, Npy1r^flox/+^*), and homozygous pancreas-specific *Npy1r* knockout KP^R172H^C mice (*Pdx1-Cre, LSL-Kras^G12D/+^, LSL-Trp53^R172H/+^, Npy1r^flox/flox^*) were generated. Furthermore, KP^R172H^C mice were crossed with *Npy1r* whole-body knockout mice (*89*, *97*) to generate cohorts of KP^R172H^C *Npy1r* wild-type mice (*Pdx1-Cre, LSL-Kras^G12D/+^, LSL-Trp53^R172H/+^, Npy1r^+/+^*), whole-body *Npy1r* heterozygous knockout KP^R172H^C mice (*Pdx1-Cre, LSL-Kras^G12D/+^, LSL-Trp53^R172H/+^, Npy1r^+/−^*), and homozygous *Npy1r* knockout KP^R172H^C mice (*Pdx1-Cre, LSL-Kras^G12D/+^, LSL-Trp53^R172H/+^, Npy1r^−/−^*). KP^R172H^C *Npy1r* WT animals were obtained from a pool of mice generated from the progeny of both genetic crosses. All mice were bred at Australian BioResources, and genotyping was performed at the Garvan Molecular Genetics facility. Mice with the appropriate genotypes were enrolled into the study at ~6 weeks of age and weighed, palpated, and monitored once weekly until detection of a palpable tumor, after which monitoring was increased to at least 3× weekly. The time to palpable tumor and time from palpable tumor to the end point were determined for all animals where tumor could be clearly detected via palpation. Mice were euthanized upon reaching the study end point, which included the development of signs of ascites, overnight weight loss of ≥10% or weight loss of ≥20% compared to the maximum body weight, hunching posture, and signs of pain. Tissues and organs were harvested from end-point animals and weighed unless determined not feasible (e.g., because of tissues/organs excessively infiltrated/invaded by the tumor). Mice were censored from the study if they had to be euthanized because of unspecific end points not related to PC, including presentation of severe prolapses, papilloma, and lymphomas.

### RNA-seq of KP^R172H^C tumors

Tumors were isolated from KP^R172H^C *Npy1r* WT and *Npy1r^−/−^* mice at the end stage, snap frozen, and stored at −80°C. RNA extraction was performed on 10 KP^R172H^C *Npy1r* WT and 10 KP^R172H^C *Npy1r^−/−^* end-stage tumors (from 10 animals with the highest number of liver metastases per genotype) using the QIAGEN RNeasy Mini Kit (no. 74104) according to the manufacturer’s instructions. RNA concentration and integrity were assessed using the Agilent 4200 TapeStation system and a Qubit 3.0 Fluorometer (Thermo Fisher Scientific). Library preparation was performed using the Illumina Stranded mRNA Library Preparation Kit according to the manufacturer’s protocol, and paired-end sequencing was performed using the NovaSeq S4 Flow Cell (300 cycles) Standard.

RNA-seq data were processed using Nextflow pipeline nf-core/rnaseq (3.16.0) (*98*). Briefly, sequence reads were aligned to mouse reference genome assembly GRCm39 using STAR (2.7.10a) (*99*) and expression counts estimated for Gencode basic gene annotation M35 using RSEM (1.3.1) (*100*). Downstream analysis was performed in R (4.4.2) using Bioconductor packages DESeq2 (1.44.0) (*101*) for differential expression analysis and fgsea (1.30.0) for GSEA. *P* values were adjusted using the Benjamini-Hochberg procedure to control the false discovery rate.

### MS proteomics sample preparation

Tumors were isolated from KP^R172H^C *Npy1r* WT and *Npy1r^−/−^* at the end stage, snap frozen, and stored at −80°C. Samples were prepared as previously reported (*19*). Briefly, samples were lysed with a bead beater in 1% SDS and 100 mM tris (pH 8.5) for 2 min at 50 Hz. Cell debris was removed by centrifugation, and the supernatant was denatured with 100 mM tris(2-carboxyethyl)phosphine hydrochloride and alkylated with 40 mM chloroacetic acid for 5 min at 45°C. Protein concentration was determined using Qubit (Protein Assay Kit, Life Technologies). Proteins were coupled to a 1:1 mix of hydrophilic:hydrophobic Sera-Mag SpeedBeads (Cytvia) as per the manufacturer’s instructions at a ratio of 1 μg of protein:10 μg of beads with incubation at room temperature for 8 min. Beads were washed three times with 80% ethanol, dried, and resuspended in 100 μl of 10% tetrafluoroethylene in 100 mM tris-HCl (pH 7.5). Proteins were then digested with the addition of 1:50 sequencing-grade trypsin (Sigma-Aldrich) and 1:50 LysC sequencing grade LysC (Wako, Japan) (micrograms of protease:micrograms of protein) overnight at 37°C and 600 rpm in a ThermoMixer. Digestion was stopped by acidification to 1% trifluoroacetic acid (TFA), and peptides were desalted using SDB-RPS (styrenedivinylbenzene reverse phase sulfonate) StageTips. Briefly, StageTips were equilibrated with 100% acetonitrile (ACN), 30% methanol/1% TFA, and 0.2% TFA. Peptides were added and then washed with 99% ethyl acetate and 1% TFA. Peptides were eluted with 5% ammonium hydroxide/80% ACN. Peptides were dried for 1 hour at 45°C in a Speedvac and stored at −30°C before liquid chromatography–tandem MS (LC-MS/MS) analysis.

### DIA LC-MS/MS proteomics

Peptides were reconstituted in 3% ACN and 0.1% formic acid (FA). One microgram of peptide was separated on an in-house packed column (150 μm by 150 mm, 1.9-μm ReproSil Pur 120 C18, Dr. Maisch GmbH, Germany) with a Vanquish Neo Liquid Chromatography (Thermo Fisher Scientific) with mobile phases A [0.1% (v/v) FA] and B [80% (v/v) ACN and 0.1% (v/v) FA]. Peptides were eluted at a flowrate of 1.2 μl/min and a gradient of 5% B to 35% B in 35 min, 35% B to 60% B in 1 min, and 60% B to 98% B in 30 s and then washed with 95% B for 3 min. The LC was coupled to an Orbitrap Exploris 480 mass spectrometer (Thermo Fisher Scientific) with a spray voltage of 2600 V, a radio frequency lens of 30%, and an ion transfer tube heated to 320°C. The Orbitrap Exploris 480 was operated in data-independent acquisition (DIA) mode as previously reported (*19*, *102*) comprising of a survey MS scan acquired as a profile with a maximum injection time of 54 ms and a standard automatic gain control target across a scan range from 350 to 1650 *m*/*z* with an Orbitrap resolution of 30,000. For MS/MS analysis, peptides were isolated with 34 variable width overlapping windows for a normalized automatic gain control target of a 1000% maximum isolation time of 30 ms (Table 3). These were fragmented with stepped higher-energy collisional dissociation collision energies of 25, 27, and 30 and analyzed across a mass range from 300 to 2000 *m*/*z* with an Orbitrap resolution of 15,000. Data files were searched with Spectronaut version 19.1 using a *Mus musculus* database (UniProt release 2024_04) and Trypsin and Lys-C/P enzymatic cleavage rules. The default settings were used for the remaining parameters. Raw data were normalized using log_2_(*x*) transformation and median subtraction. To assess for differentially abundant candidate proteins between two conditions, filters for >1.25-fold change and a *q* value (adjusted *P* value) of <0.05 were applied. GSEA analysis was performed using fgsea (1.30.0).

**Table 3.** Quadrupole isolation windows used for DIA MS analysis.

Window number	Window start (*m*/*z*)	Window end (*m*/*z*)
1	350	375
2	374	397
3	396	413
4	412	428
5	427	442
6	441	455
7	454	468
8	467	480
9	479	492
10	491	505
11	504	518
12	517	532
13	531	546
14	545	560
15	559	574
16	573	589
17	588	604
18	603	620
19	619	636
20	635	653
21	652	670
22	669	688
23	687	707
24	706	728
25	727	749
26	748	776
27	775	803
28	802	834
29	833	865
30	864	904
31	903	963
32	962	1032
33	1031	1101
34	1100	1650

### Cell culture

Primary KP^R172H^C (TB32043) (*11*, *83*, *84*) and KP^flox^C cancer cells (*16*, *35*) were previously isolated from the tumors of end-stage KP^R172H^C mice (*Pdx1-Cre, LSL-Kras^G12D/+^, LSL-Trp53^R172H/+^*) and KP^flox^C mice (*Pdx1-Cre, LSL-Kras^G12D/+^, Trp53^flox/+^*) (*16*). Telomerase-immortalized fibroblasts (TIFs) were also generated previously (*103*). Both cancer cell lines and TIFs were cultured in Dulbecco’s modified Eagle’s medium (DMEM; high glucose, pyruvate, Gibco) supplemented with 10 mM Hepes (Thermo Fisher Scientific), 10% fetal bovine serum (HyClone), and 1% penicillin/streptomycin (Thermo Fisher Scientific) at 37°C with 20% O_2_ and 5% CO_2_. All cell lines were regularly tested for mycoplasma (all negative results).

### Cell-derived matrices (CDMs)

CDMs were generated as described previously (*85*, *86*). Before CDM generation, the glass surface was coated with 0.2% gelatin and allowed to set at 37°C for 2 hours before being rinsed twice with Dulbecco’s PBS and formalin fixed for 30 min at room temperature. After a further two washes with PBS, the fixed gelatin cross-links were quenched in 1 M sterile glycine for 30 min at room temperature, and coated plates were rinsed twice with PBS and once with DMEM before TIF cell seeding. To generate the CDMs, on day 0, 1.5 × 10^5^ TIFs were seeded per well in a glass-bottom 24-well plate (Corning). On days 1, 3, and 5, cells were treated with ascorbic acid (50 μg/ml) in DMEM supplemented with 10 mM Hepes, 10% fetal bovine serum, and 1% penicillin/streptomycin. On day 7, the CDMs were denuded, using an extraction buffer [0.5% (v/v) Triton X-100, 20 mM NH_4_OH, and 0.01% sodium deoxycholate, made up in PBS], and rinsed twice with PBS, before addition of deoxyribonuclease I (10 μg/ml; Roche) in PBS containing calcium and magnesium, to digest DNA residue. This was followed by two PBS rinses before 4000 TB32043 KP^R172H^C or 4000 KP^flox^C cancer cells were seeded and allowed to adhere and grow for 16 hours. BIBO3304 (1 μM) or vehicle (water) was added to the cells before subsequent live cell imaging for 8 hours using a Leica Live Cell system with a Leica DMi6000 inverted microscope or an IncuCyte S3 Live-Cell Analysis Instrument (Sartorius). Tracking and quantification of cell migration were performed on binary images using a MATLAB plug-in (CellTracker) (*104*).

### Intrasplenic injections

For intrasplenic experiments, 5 × 10^5^ TB32043 KP^R172H^C cells were injected into the spleens of C57BL/6JAusb mice in 50 μl of Hanks Balanced Salt Solution as previously described (*33*, *90*). Mice were first anesthetized using gaseous isoflurane, and a left subcostal incision was made through the skin. The cell suspension was then injected into the spleen during open laparotomy, followed by application of cyanoacrylate to the injection site to prevent hemorrhage from the spleen. The surgery site was closed using 5-mm resorbable vicryl sutures on the peritoneal wall and clips for the skin, and mice were treated with analgesia bupivacaine (topically, 8 mg/kg) and buprenorphine (subcutaneously, 0.075 mg/kg). BIBO3304 (1 mg/kg) or vehicle (saline) was administered daily via intraperitoneal injection (day 0 to day 11). Mice were euthanized either on day 5 or on day 12, and the number of visible metastases in the liver was quantified by first separating the liver into its various lobes and then counting the number of metastases on the front and back on the lobes. Livers were subsequently fixed in formalin, dehydrated, and paraffin embedded for histological analysis. Animals with surgical complications were euthanized immediately and excluded from the study.

## Supplementary Materials

### The PDF file includes:

Figs. S1 to S9 Legends for tables S1 and S2 Legends for movies S1 and S2 Consortium members of the Australian Pancreatic Cancer Genome Initiative (APGI) Consortium members of the Avner Australian Pancreatic Cancer Matrix Atlas (APMA)
