# Supplementary material for: Targeting the NPY/NPY1R signaling axis in mutant p53–dependent pancreatic cancer impairs metastasis
Source: Sci Adv. 2025 Mar 12;11(11):eadq4416. doi: 10.1126/sciadv.adq4416 (PMC11900870; doi:10.1126/sciadv.adq4416)
Supplement: Supplementary file 1 — Figs. S1 to S9 Legends for tables S1 and S2 Legends for movies S1 and S2 Consortium members of the Australian Pancreatic Cancer Genome Initiative (APGI) Consortium members of the Avner Australian Pancreatic Cancer Matrix Atlas (APMA) [file sciadv.adq4416_sm.pdf]

Science Advances
AAAS

Supplementary Materials for

Targeting the NPY/NPY1R signaling axis in mutant p53–dependent pancreatic cancer impairs metastasis

Cecilia R. Chambers *et al.*

Corresponding author: Daniel Christ, d.christ@garvan.org.au; Herbert Herzog, h.herzog@unsw.edu.au; Paul Timpson, p.timpson@garvan.org.au; David Herrmann, d.herrmann@garvan.org.au

Sci. Adv. **11**, eadq4416 (2025)
DOI: 10.1126/sciadv.adq4416

The PDF file includes:

Figs. S1 to S9
Legends for tables S1 and S2
Legends for movies S1 and S2
Consortium members of the Australian Pancreatic Cancer Genome Initiative (APGI)
Consortium members of the Avner Australian Pancreatic Cancer Matrix Atlas (APMA)

Other Supplementary Material for this manuscript includes the following:

Tables S1 and S2
Movies S1 and S2

Fig. S1.

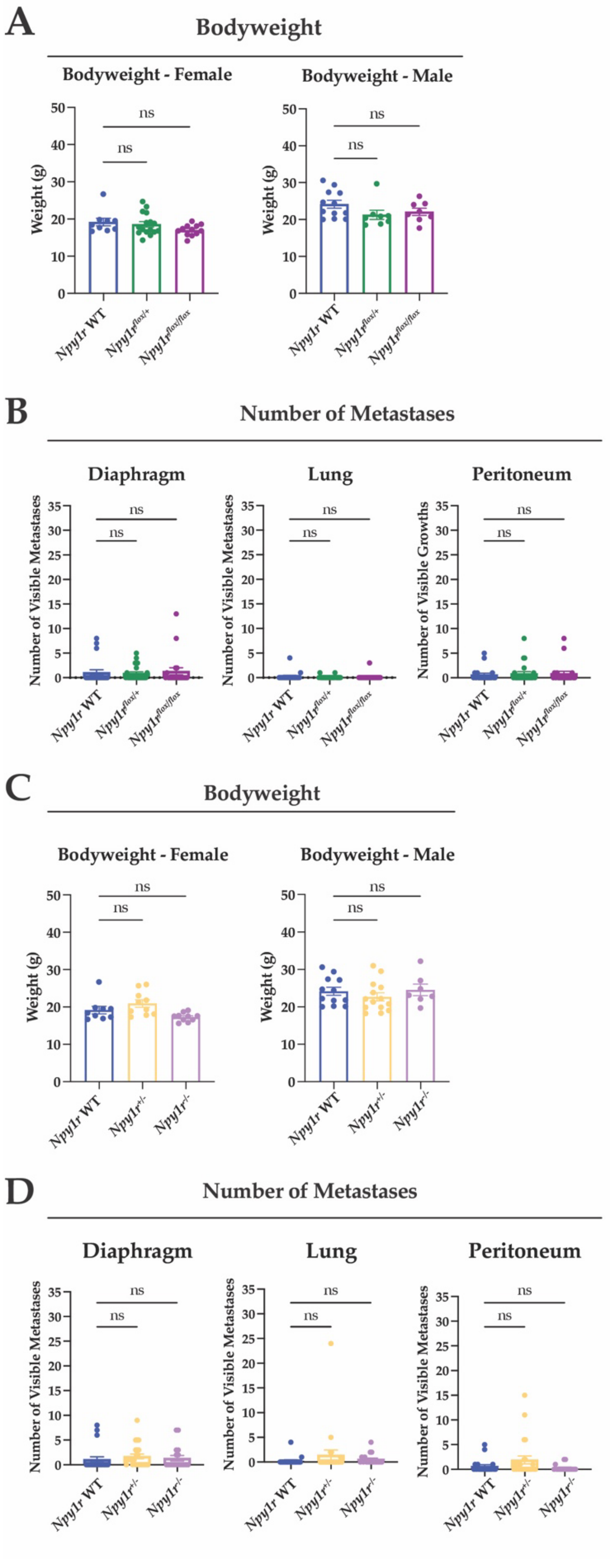
A
Bodyweight
Bodyweight - Female
Bodyweight - Male
Weight (g)
ns
Npy1r WT
Npy1r flox/+
Npy1r flox/flox
B
Number of Metastases
Diaphragm
Lung
Peritoneum
Number of Visible Metastases
Number of Visible Growths
ns
Npy1r WT
Npy1r flox/+
Npy1r flox/flox
C
Bodyweight
Bodyweight - Female
Bodyweight - Male
Weight (g)
ns
Npy1r WT
Npy1r +/-
Npy1r -/-
D
Number of Metastases
Diaphragm
Lung
Peritoneum
Number of Visible Metastases
ns
Npy1r WT
Npy1r +/-
Npy1r -/-

Fig. S1. *Npy1r* conditional and whole-body knockout in KP^{R172H}C mice results in no alteration in bodyweight or metastasis to non-liver organs. (A) Quantification of bodyweight separated by sex of pancreas-specific *Npy1r* KP^{R172H}C knockout mice at study endpoint. **(B)** Quantification of visible metastases in the lung, diaphragm, and peritoneum of pancreas-specific *Npy1r* KP^{R172H}C knockout mice at study endpoint. **(C)** Quantification of bodyweight separated by sex of whole-body *Npy1r* KP^{R172H}C knockout mice at study endpoint. **(D)** Quantification of visible metastases in the lung, diaphragm, and peritoneum of whole-body *Npy1r* KP^{R172H}C knockout mice at study endpoint. Data for *Npy1r* WT was the same for both pancreas-specific and whole-body *Npy1r* knockout survival studies. *Npy1r* WT data on body weight are shown in Fig. S1A, and again in S1C. *Npy1r* WT data on number of metastases are shown in Fig. S1B, and again in S1D. Mean ± SEM. ns, $P \geq 0.05$ by a one-way ANOVA.

Fig. S2.

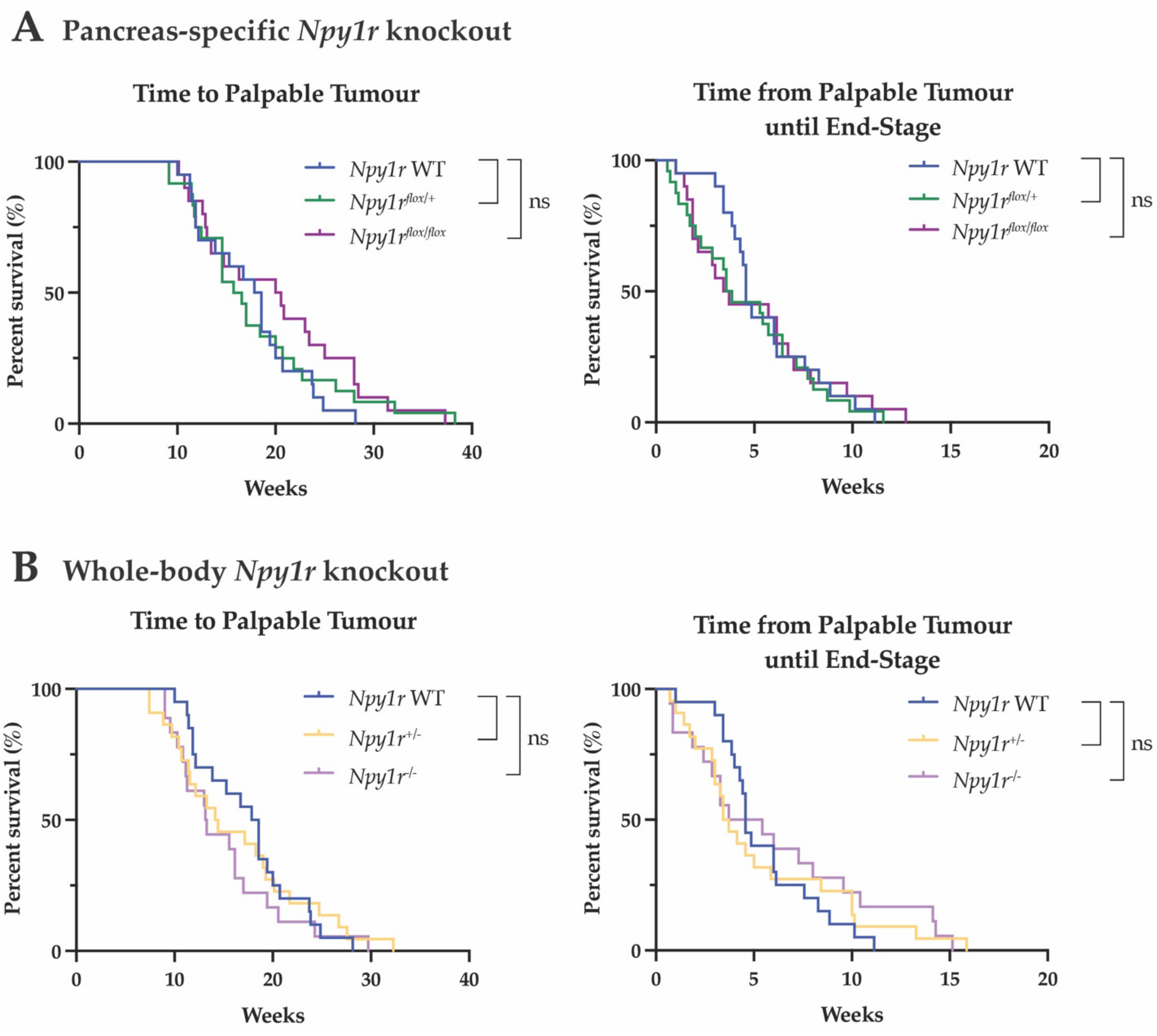

Fig. S2. *Npy1r* conditional and whole-body knockout in KP^{R172H}C mice does not alter time to palpable tumor or time from palpable tumor to end-stage. (A) Kaplan-Meier analysis of time to palpable tumor and time from palpable tumor to end-stage following pancreas-specific *Npy1r* knockout in KP^{R172H}C mice for the three different genotypes *Npy1r* WT, *Npy1r*$^{flox/+}$, *Npy1r*$^{flox/flox}$ (n ≥ 20 mice per genotype). **(B)** Kaplan-Meier analysis of time to palpable tumor and time from palpable tumor to end-stage following whole-body *Npy1r* knockout in KP^{R172H}C mice for the three different genotypes *Npy1r* WT, *Npy1r*$^{+/-}$, *Npy1r*$^{-/-}$ (n ≥ 18 mice per genotype). Data for *Npy1r* WT was the same for both pancreas-specific and whole-body *Npy1r* knockout survival studies and is shown in Fig. S2A, and again in S2B. ns, P ≥ 0.05 by Kaplan-Meier survival analysis.

Fig. S3.

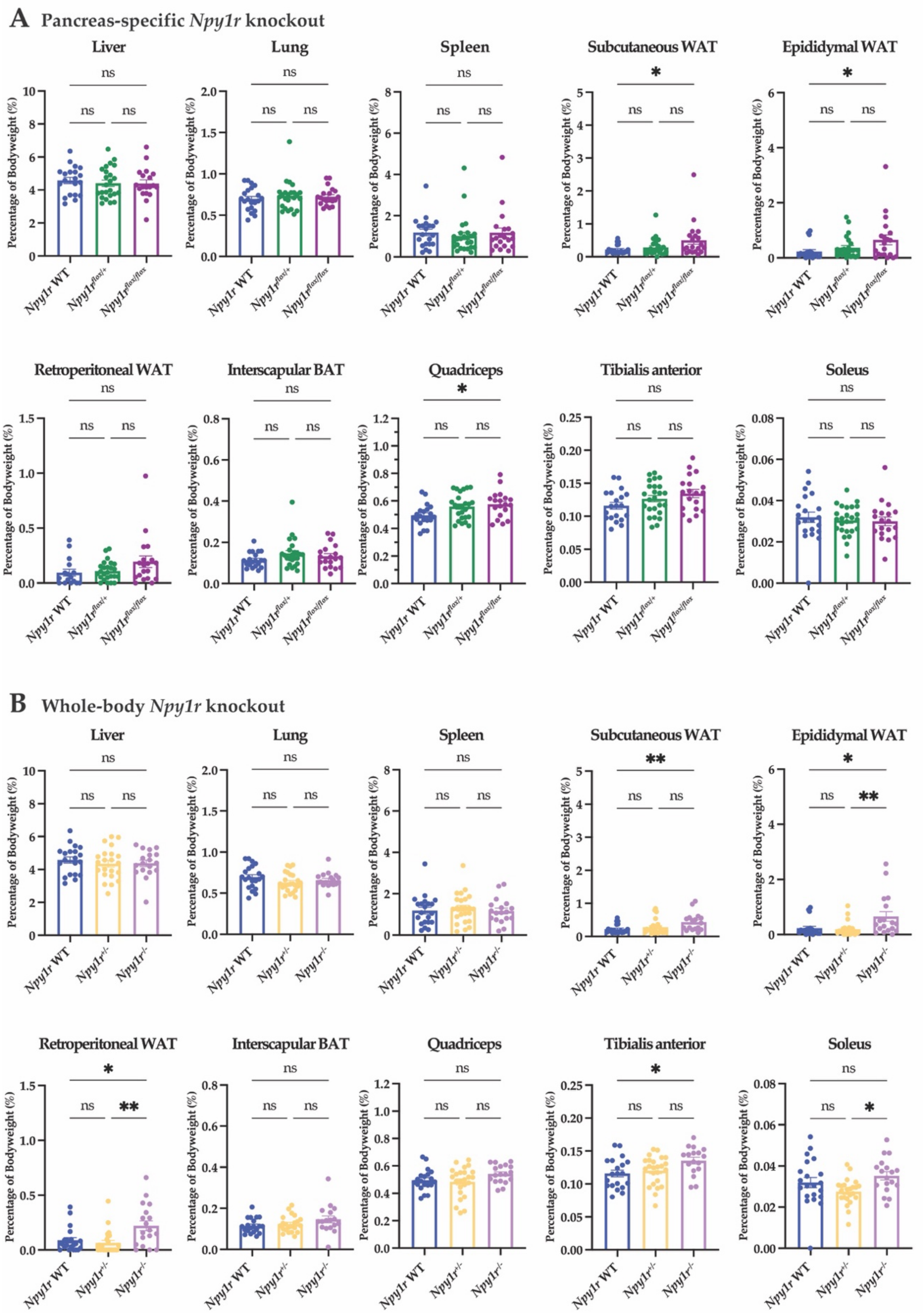
A Pancreas-specific Npy1r knockout
Liver
Lung
Spleen
Subcutaneous WAT
Epididymal WAT
Retroperitoneal WAT
Interscapular BAT
Quadriceps
Tibialis anterior
Soleus
Percentage of Bodyweight (%)
ns
*
Npy1r WT
Npy1r flox/+
Npy1r flox/flox
B Whole-body Npy1r knockout
**
Npy1r +/-
Npy1r -/-

Fig. S3. Tissue and organ weights in *Npy1r* conditional and whole-body knockout in KP^{R172H}C mice. (A) Quantification of tissue and organ weights as percentage of bodyweight isolated from pancreas-specific *Npy1r* KP^{R172H}C knockout mice at study endpoint (n ≥ 16 per genotype). **(B)** Quantification of tissue and organ weights as percentage of bodyweight isolated from whole-body *Npy1r* KP^{R172H}C knockout mice at study endpoint (n ≥ 17 per genotype). Data for *Npy1r* WT was the same for both pancreas-specific and whole-body *Npy1r* knockout survival studies and are shown in Fig. S3A, and again in S3B. WAT, white adipose tissue. BAT, brown adipose tissue. Mean ± SEM. *, P < 0.05; **, P < 0.01; ns, P ≥ 0.05 by a one-way ANOVA.

Fig. S4.

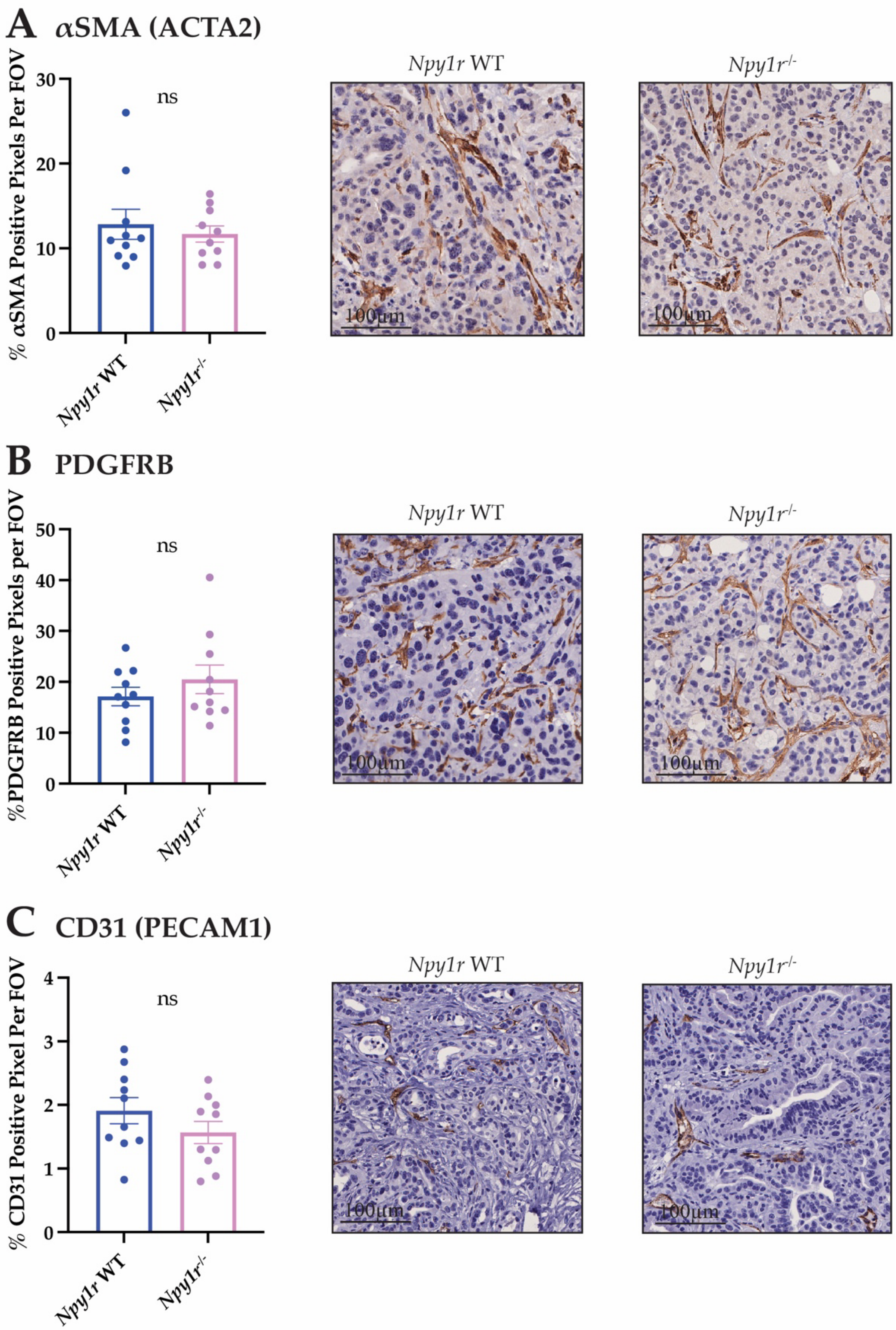
A
αSMA (ACTA2)
% αSMA Positive Pixels Per FOV
ns
Npy1r WT
Npy1r-/-
100µm
B
PDGFRB
% PDGFRB Positive Pixels per FOV
ns
Npy1r WT
Npy1r-/-
100µm
C
CD31 (PECAM1)
% CD31 Positive Pixel Per FOV
ns
Npy1r WT
Npy1r-/-
100µm

Fig. S4. *Npy1r* whole-body knockout in $KP^{R172H}C$ mice does not affect CAF or endothelial cell abundance. (A) Quantification and representative images of αSMA (ACTA2) immunohistochemistry in *Npy1r* WT and $Npy1r^{-/-}$ $KP^{R172H}C$ end-stage tumors (n = 10 per genotype). **(B)** Quantification and representative images of PDGFRB immunohistochemistry in *Npy1r* WT and $Npy1r^{-/-}$ $KP^{R172H}C$ end-stage tumors (n = 10 per genotype). **(C)** Quantification and representative images of CD31 (PECAM1) immunohistochemistry in *Npy1r* WT and $Npy1r^{-/-}$ $KP^{R172H}C$ end-stage tumors (n = 10 per genotype). Scale bar, 100μm. Mean ± SEM. ns, $P \geq 0.05$ by an unpaired parametric t test.

Fig. S5.

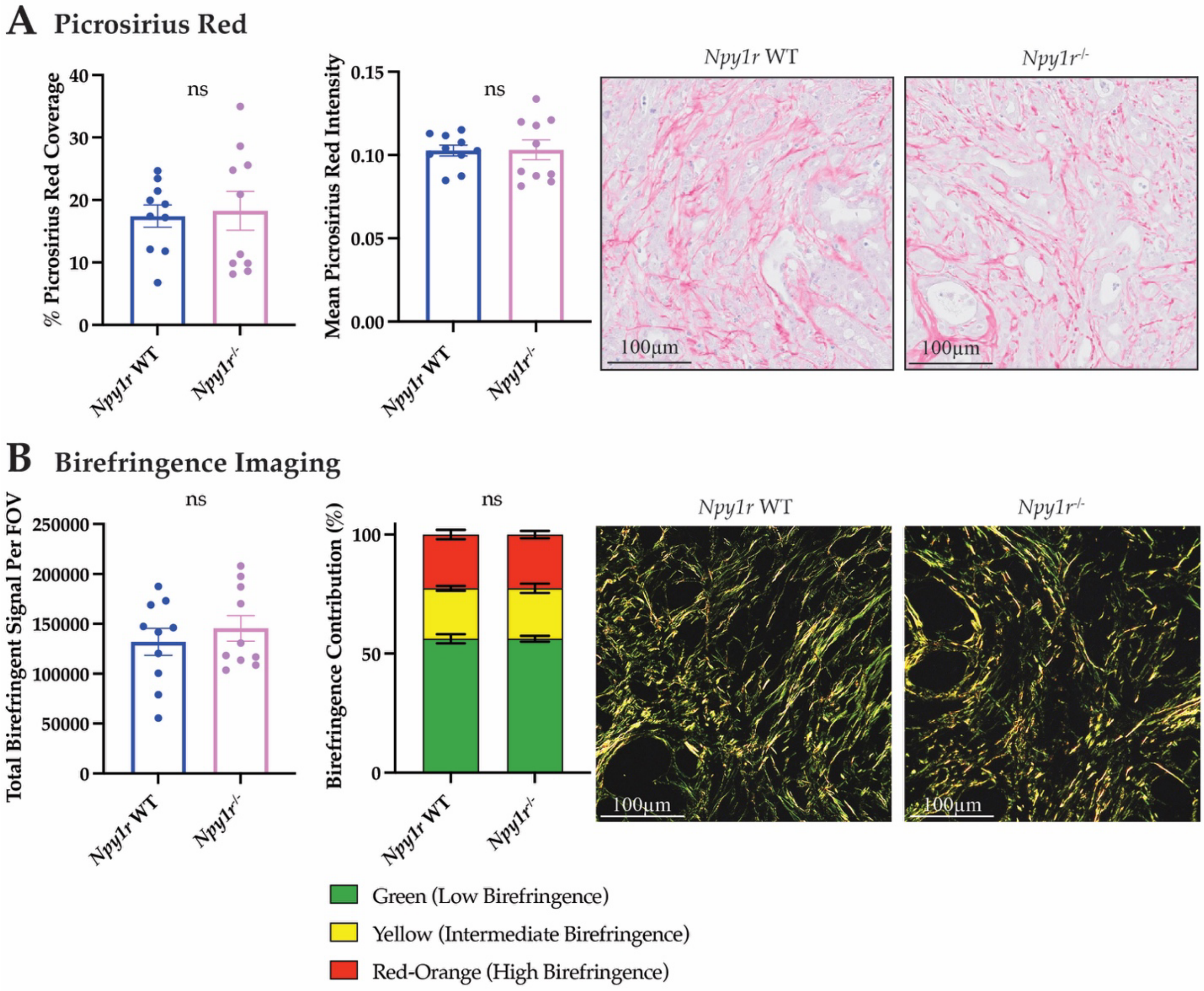

Fig. S5. ***Npy1r*** **whole-body knockout in KP^{R172H}C mice does not affect fibrillar collagen abundance assessed by Picrosirius Red histological staining. (A)** Quantification of Picrosirius Red coverage and intensity with representative transmitted light images of *Npy1r* WT and *Npy1r*$^{-/-}$ KP^{R172H}C end-stage tumors (n = 10 per genotype). **(B)** Quantification of total birefringence signal and contribution of high (red), medium (yellow) and low (green) birefringence to the total birefringent signal (indicative of a range from highly mature to nascent fibers, respectively) with representative polarized light images of *Npy1r* WT and *Npy1r*$^{-/-}$ KP^{R172H}C end-stage tumors (n = 10 per genotype). Scale bar, 100µm. Mean ± SEM. ns, P ≥ 0.05 by an unpaired parametric t test (**A**, **B** Total Birefringent Signal) or a two-way ANOVA (**B**, Birefringence Contribution).

Fig. S6.

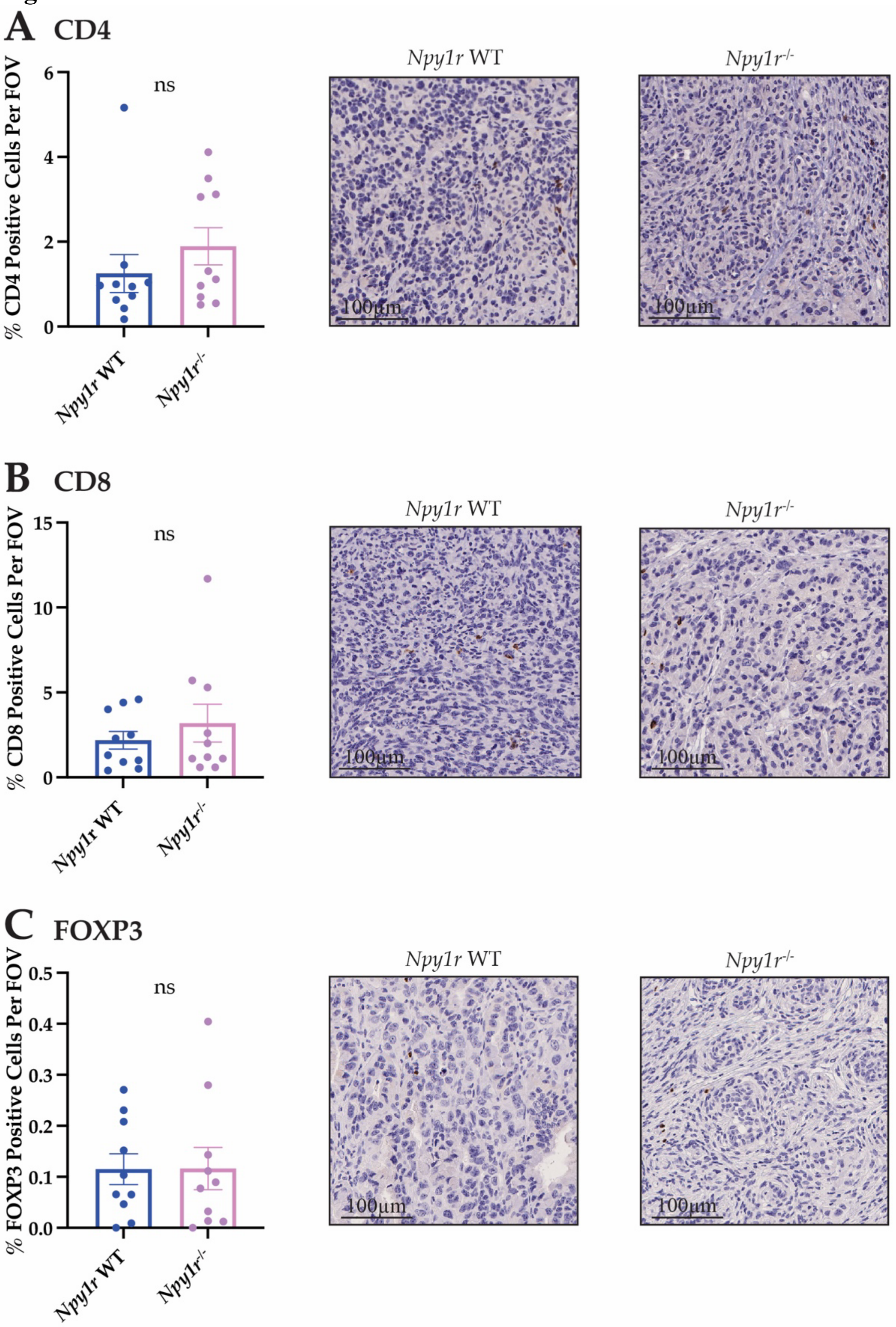

Fig. S6. *Npy1r* whole-body knockout in KP^{R172H}C mice does not affect immune cell abundance. (A) Quantification and representative images of CD4 immunohistochemistry in

Npy1r WT and *Npy1r*$^{-/-}$ KP^{R172H}C end-stage tumors (n = 10 per genotype). **(B)** Quantification and representative images of CD8 immunohistochemistry in *Npy1r* WT and *Npy1r*$^{-/-}$ KP^{R172H}C end-stage tumors (n = 10 per genotype). **(C)** Quantification and representative images of FOXP3 immunohistochemistry in *Npy1r* WT and *Npy1r*$^{-/-}$ KP^{R172H}C end-stage tumors (n = 10 per genotype). Scale bar, 100µm. Mean ± SEM. ns, $P \geq 0.05$ by an unpaired parametric t test.

Fig. S7.

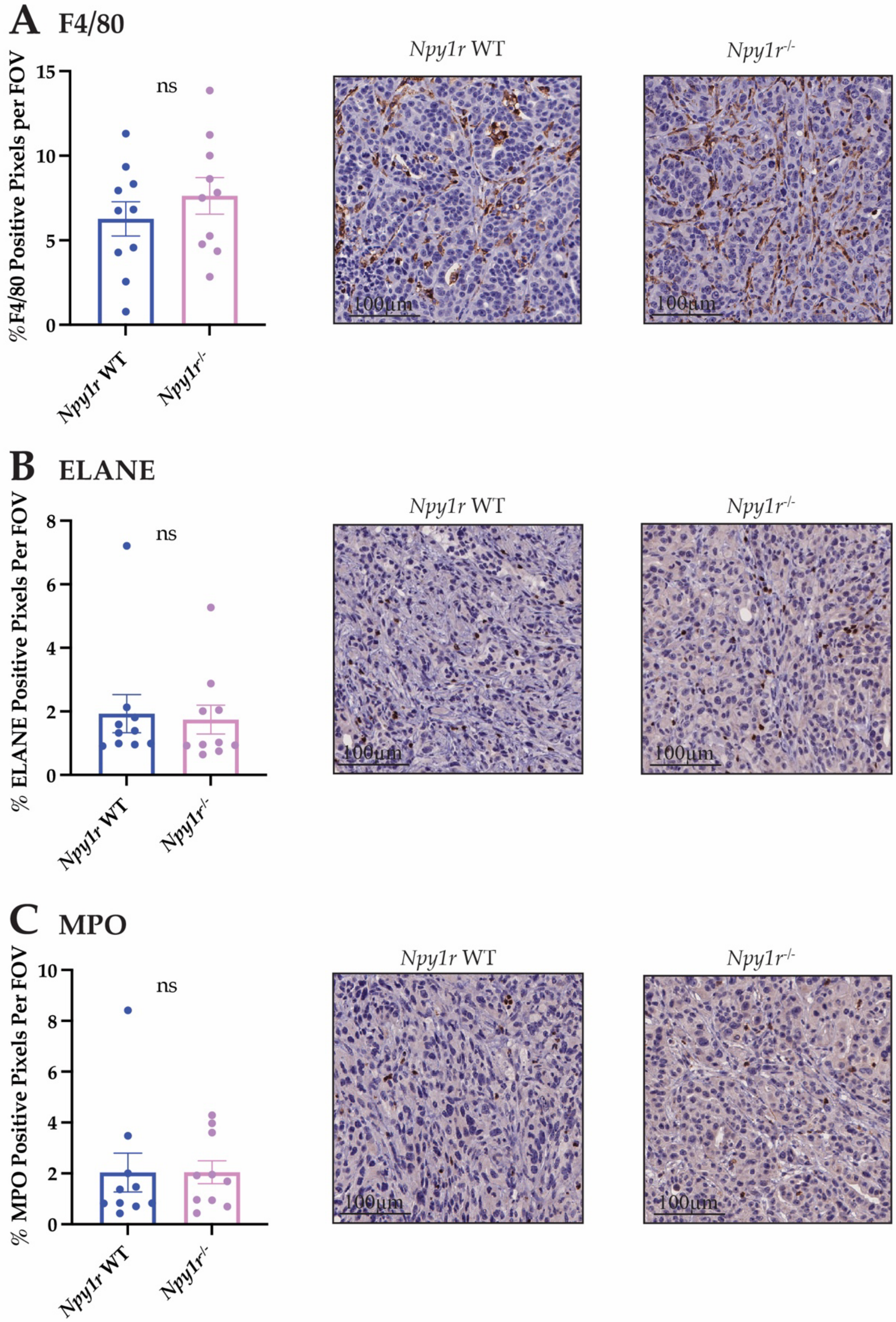

Fig. S7. *Npy1r* whole-body knockout in KP^{R172H}C mice does not affect immune cell abundance. (A) Quantification and representative images of F4/80 immunohistochemistry in

Npy1r WT and *Npy1r*$^{-/-}$ KP^{R172H}C end-stage tumors (n = 10 per genotype). **(B)** Quantification and representative images of ELANE immunohistochemistry in *Npy1r* WT and *Npy1r*$^{-/-}$ KP^{R172H}C end-stage tumors (n = 10 per genotype). **(C)** Quantification and representative images of MPO immunohistochemistry in *Npy1r* WT and *Npy1r*$^{-/-}$ KP^{R172H}C end-stage tumors (n = 10 per genotype). Scale bar, 100µm. Mean ± SEM. ns, $P \geq 0.05$ by an unpaired parametric t test.

Fig. S8.

A RNA-seq: Volcano Plot comparing mRNA expression between *Npy1r* WT and *Npy1r*$^{-/-}$ end-stage tumors

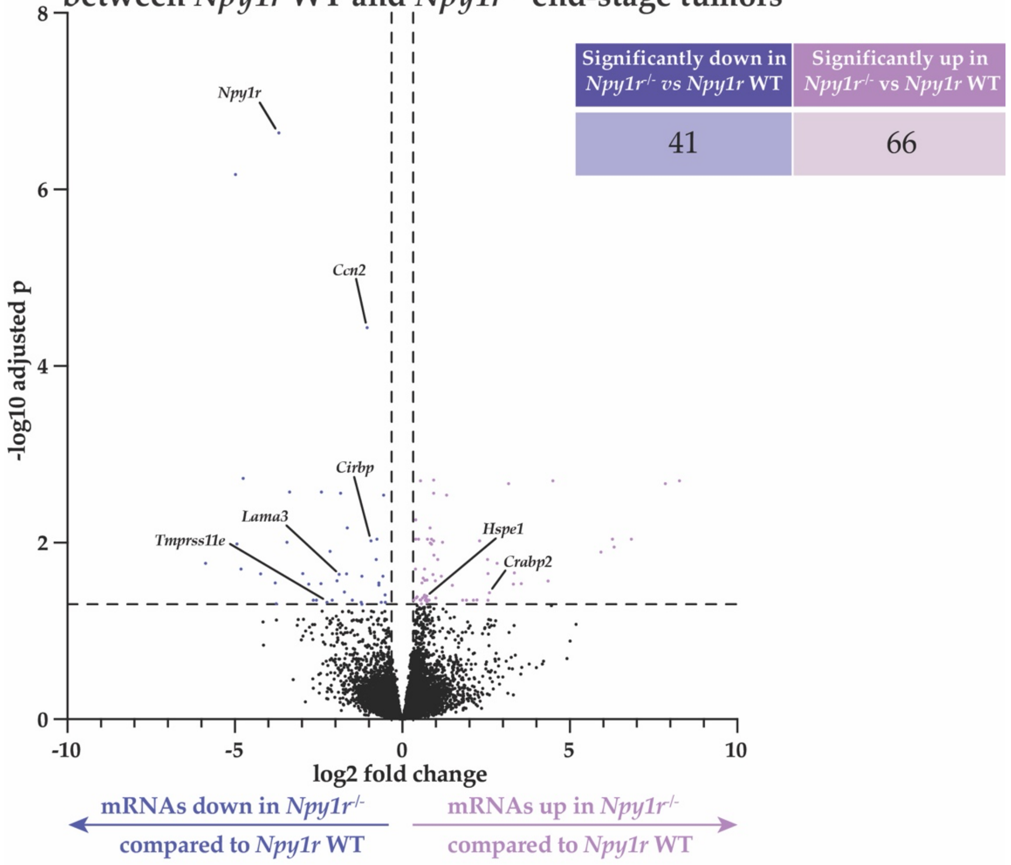

B RNA-seq: GSEA analysis

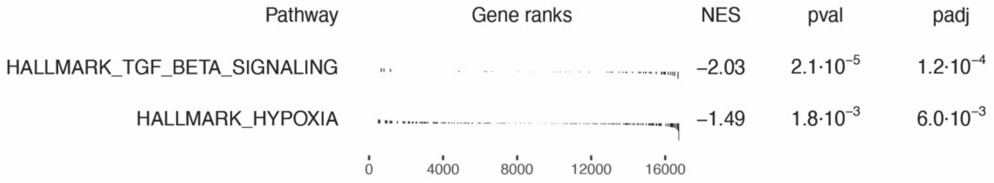

pathway	pval	padj	log2err	ES	NES
HALLMARK_TGF_BETA_SIGNALING	2.13E-05	0.0001182	0.57561026	-0.5839464	-2.0337774
HALLMARK_ESTROGEN_RESPONSE_EARLY	5.20E-05	0.00023635	0.55733224	-0.3924107	-1.6601633
HALLMARK_APICAL_JUNCTION	0.00027581	0.00106081	0.49849311	-0.3702428	-1.5672255
HALLMARK_HYPOXIA	0.0018071	0.00602368	0.45505987	-0.3523679	-1.4933223
HALLMARK_INFLAMMATORY_RESPONSE	0.01089382	0.03204064	0.158178	0.36969688	1.39877465
HALLMARK_UNFOLDED_PROTEIN_RESPONSE	0.00837592	0.02617476	0.18806904	0.41677081	1.47472653
HALLMARK_INTERFERON_ALPHA_RESPONSE	0.00163569	0.00584174	0.45505987	0.46811026	1.61199728
HALLMARK_INTERFERON_GAMMA_RESPONSE	0.00011343	0.00047261	0.5384341	0.43448941	1.64222685
HALLMARK_FATTY_ACID_METABOLISM	3.44E-05	0.00017191	0.55733224	0.4733976	1.74056347
HALLMARK_ALLOGRAFT_REJECTION	3.42E-07	2.14E-06	0.67496286	0.49559849	1.85875027
HALLMARK_MTORC1_SIGNALING	2.43E-07	1.74E-06	0.67496286	0.49297584	1.87548061
HALLMARK_DNA_REPAIR	1.00E-09	1.00E-08	0.78818681	0.56515942	2.08056165
HALLMARK_MYC_TARGETS_V2	4.53E-08	3.78E-07	0.71951283	0.67955942	2.18102791
HALLMARK_OXIDATIVE_PHOSPHORYLATION	1.94E-17	2.43E-16	1.07686819	0.62232259	2.36536281
HALLMARK_G2M_CHECKPOINT	4.46E-23	7.44E-22	1.24623277	0.66679048	2.53437918
HALLMARK_MYC_TARGETS_V1	2.83E-28	7.08E-27	1.38026492	0.70102339	2.66449378
HALLMARK_E2F_TARGETS	5.86E-33	2.93E-31	1.49547933	0.72295695	2.75394771

Fig. S8. Gene expression changes on transcriptional level in primary tumors isolated from $KP^{R172H}C$ *Npy1r*$^{-/-}$ mice compared to $KP^{R172H}C$ *Npy1r* WT mice. (A) Volcano plot depicting significantly down-regulated (blue) and significantly up-regulated transcripts (purple) in $KP^{R172H}C$ *Npy1r*$^{-/-}$ compared to $KP^{R172H}C$ *Npy1r* WT tumors (n = 10 per genotype, adjusted p < 0.05 [horizontal line], > 1.25 fold change [vertical lines]). **(B)** Gene Set Enrichment Analysis showing significantly de-regulated Hallmarks pathways in $KP^{R172H}C$ *Npy1r*$^{-/-}$ compared to $KP^{R172H}C$ *Npy1r* WT tumors (n = 10 per genotype, adjusted p < 0.05).

Fig. S9.

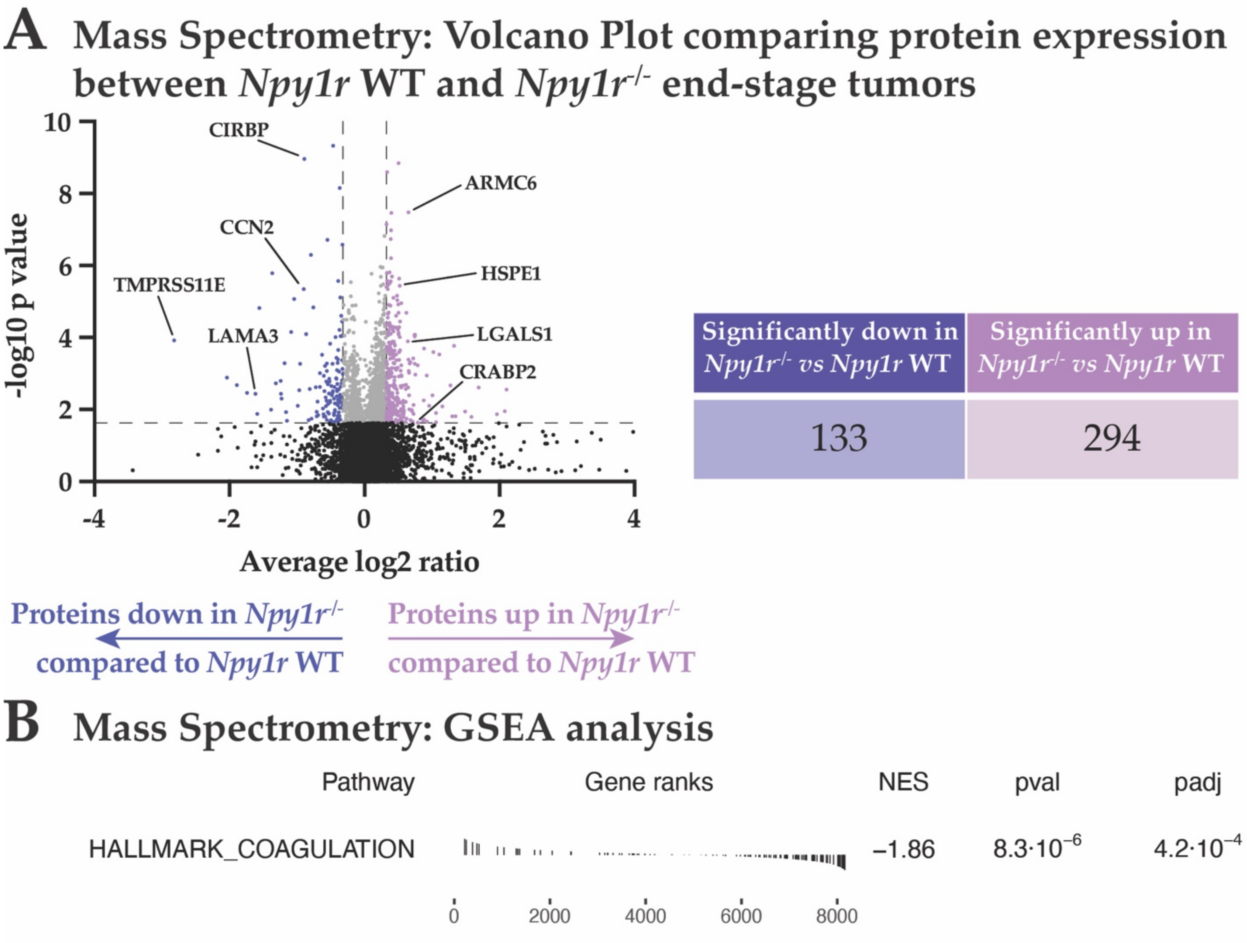

pathway	pval	padj	log2err	ES	NES
HALLMARK_COAGULATION	8.32E-06	0.00041601	0.59332548	-0.4525506	-1.8568247
HALLMARK_COMPLEMENT	0.00188227	0.03137111	0.45505987	-0.3628177	-1.5205533
HALLMARK_OXIDATIVE_PHOSPHORYLATION	3.34E-05	0.00083576	0.55733224	0.46865361	1.66768637

Fig. S9. Gene expression changes on proteomic level in primary tumors isolated from KP^{R172H}C *Npy1r$^{-/-}$* mice compared to KP^{R172H}C *Npy1r* WT mice. (A) Volcano plot depicting significantly down-regulated (blue) and significantly up-regulated proteins (purple) in KP^{R172H}C *Npy1r$^{-/-}$* compared to KP^{R172H}C *Npy1r* WT tumors (n = 10 per genotype, q < 0.05 [horizontal lines], > 1.25 fold change [vertical lines]). **(B)** Gene Set Enrichment Analysis showing significantly de-regulated Hallmarks pathways in KP^{R172H}C *Npy1r$^{-/-}$* compared to KP^{R172H}C *Npy1r* WT tumors (n = 10 per genotype, adjusted p < 0.05).

Table S1.

Differentially expressed transcripts determined by RNAseq of end-stage tumors isolated from KP^{R172H}C *Npy1r*$^{-/-}$ *versus* KP^{R172H}C *Npy1r* WT mice (n = 10 per genotype). Adjusted p value < 0.05, fold change > 1.25.

Table S2.

Differentially abundant proteins determined by mass spectrometry proteomics of end-stage tumors isolated from KP^{R172H}C *Npy1r*$^{-/-}$ *versus* KP^{R172H}C *Npy1r* WT mice (n = 10 per genotype). q value < 0.05, fold change > 1.25.

Movie S1.

Time-lapse video of $KP^{R172H}C$ cells migrating upon cell-derived matrices (CDMs) following vehicle or BIBO3304 treatment over ~8hours. Scale bar, 100µm.

Movie S2.

Time-lapse video of $KP^{flox}C$ cells migrating upon cell-derived matrices (CDMs) following vehicle or BIBO3304 treatment over ~8hours. Scale bar, 100µm.

Australian Pancreatic Cancer Genome Initiative (APGI) consortium members:

Garvan Institute of Medical Research Amber L. Johns[1], Anthony J. Gill[1,5], Lorraine A. Chantrill[1,22], Paul Timpson[1,] Angela Chou[1,5], Marina Pajic[1], Tanya Dwarte[1], David Herrmann[1], Claire Vennin[1], Thomas R. Cox[1], Brooke A. Pereira[1], Shona Ritchie[1], Daniel A. Reed[1], Cecilia R. Chambers[1], Max Nobis[1], Gloria Jeong[1], Ruth J. Lyons[1], Nicola Blackburn[1], Adnan Nagrial[1], Sean Porazinski[1], Diego Chacon Fajardo[1], Alice Russo[1]. **QIMR Berghofer Medical Research Institute** Nicola Waddell[2], John V. Pearson[2], Katia Nones[2], Felicity Newell[2], Venkateswar Addala[2], Oliver Holmes[2], Conrad Leonard[2], Scott Wood[2]. **University of Melbourne, Centre for Cancer Research** Sean M. Grimmond[3], Oliver Hofmann[3]. **Royal North Shore Hospital** Jaswinder S. Samra[5], Nick Pavlakis[5,] Jennifer Arena[5], Hilda A. High[5], Anubhav Mittal[5]. **Bankstown Hospital** Ray Asghari[6], Neil D. Merrett[6], Amitabha Das[6]. **Liverpool Hospital** Peter H. Cosman[7], Kasim Ismail[7]. **St Vincent's Hospital** Alina Stoita[8], David Williams[8], Allan Spigellman[8]**. Westmead Hospital** Duncan McLeod[9], Judy Kirk[9]. **Royal Prince Alfred Hospital, Chris O'Brien Lifehouse** James G. Kench[10], Peter Grimison[10], Charbel Sandroussi[10], Annabel Goodwin[7,10]. **Prince of Wales Hospital** R. Scott Mead[1,11], Katherine Tucker[11], Lesley Andrews[11]. **Fiona Stanley Hospital** Michael Texler[12], Cindy Forrest[12], Mo Ballal[12,13], David Fletcher[12]**. St John of God Healthcare** Maria Beilin[13], Kynan Feeney[13] Krishna Epari[13] Sanjay Mukhedkar[13]. **Epworth HealthCare** Nikolajs Zeps[23]. **Royal Adelaide Hospital** Nan Q Nguyen[14], Andrew R. Ruszkiewicz[14], Chris Worthley[14]. **Flinders Medical Centre** John Chen[15], Mark E. Brooke-Smith[15], Virginia Papangelis[15]. **Envoi Pathology** Andrew D. Clouston[16]. **Princess Alexandra Hospital** Andrew P. Barbour[17], Thomas J. O'Rourke[17], Jonathan W. Fawcett[17], Kellee Slater[17], Michael Hatzifotis[17], Peter Hodgkinson[17]. **Austin Hospital** Mehrdad Nikfarjam[18]. **Johns Hopkins Medical Institutes** James R. Eshleman[19], Ralph H. Hruban[19], Christopher L. Wolfgang[19]. **ARC-Net Centre for Applied Research on Cancer** Aldo Scarpa[20], Rita T. Lawlor[20], Vincenzo Corbo[20], Claudio Bassi[20]. **University of Glasgow** Andrew V Biankin [21], Nigel B. Jamieson[21] David K. Chang[1, 21,] Stephan B. Dreyer[21].

[1]The Kinghorn Cancer Centre, Garvan Institute of Medical Research, 370 Victoria Street, Darlinghurst, Sydney, New South Wales 2010, Australia.
[2]QIMR Berghofer Medical Research Institute, 300 Herston Rd, Herston, Queensland 4006, Australia.
[3]University of Melbourne, Centre for Cancer Research, Victorian Comprehensive Cancer Centre, 305 Grattan Street, Melbourne, Victoria 3000, Australia.
[4]Institute for Molecular Bioscience, University of QLD, St Lucia, Queensland 4072, Australia.
[5]Royal North Shore Hospital, Westbourne Street, St Leonards, New South Wales 2065, Australia.
[6]Bankstown Hospital, Eldridge Road, Bankstown, New South Wales 2200, Australia.
[7]Liverpool Hospital, Elizabeth Street, Liverpool, New South Wales 2170, Australia.
[8] St Vincent's Hospital, 390 Victoria Street, Darlinghurst, New South Wales, 2010 Australia.
[9]Westmead Hospital, Hawkesbury and Darcy Roads, Westmead, New South Wales 2145, Australia.

[10]Royal Prince Alfred Hospital, Missenden Road, Camperdown, New South Wales 2050, Australia.
[11]Prince of Wales Hospital, Barker Street, Randwick, New South Wales 2031, Australia.
[12]Fiona Stanley Hospital, 11 Robin Warren Dr, Murdoch WA 6150, Australia
[13] St John of God Healthcare, 12 Salvado Road, Subiaco, Western Australia 6008, Australia.
[14] Royal Adelaide Hospital, North Terrace, Adelaide, South Australia 5000, Australia.
[15] Flinders Medical Centre, Flinders Drive, Bedford Park, South Australia 5042, Australia.
[16] Envoi Pathology, 1/49 Butterfield Street, Herston, Queensland 4006, Australia.
[17] Princess Alexandra Hospital, 199 Ipswich Rd, Woolloongabba QLD 4102
[18] Austin Hospital, 145 Studley Road, Heidelberg, Victoria 3084, Australia.
[19] Johns Hopkins Medical Institute, 600 North Wolfe Street, Baltimore, Maryland 21287, USA.
[20] ARC-NET Center for Applied Research on Cancer, University of Verona, Via dell'Artigliere, 19 37129 Verona, Province of Verona, Italy.
[21] Wolfson Wohl Cancer Research Centre, Institute of Cancer Sciences, University of Glasgow, Garscube Estate, Switchback Road, Bearsden, Glasgow, Scotland G61 1BD, United Kingdom.
[22] Wollongong Hospital, Illawarra and Shoalhaven Local Health District, Loftus Street, Wollongong NSW 2500.
[23] Epworth HealthCare, 89 Bridge Rd, Richmond VIC 3121, Australia

Avner Australian Pancreatic Cancer Matrix Atlas (APMA) consortium members:

Paul Timpson[1], Thomas R. Cox[1], Marina Pajic[1], Anthony J. Gill[1,2], Jaswinder S. Samra[1,2], Brooke A. Pereira[1], David Herrmann[1], Amber L. Johns[1], Gloria Jeong[1], Shona Ritchie[1], Daniel A. Reed[1], Cecilia R. Chambers[1], Janett Stoehr[1], Morghan C. Lucas[1], Joanna N. Skhinas[1], Lea Abdulkhalek[1], Max Nobis[1], Tatjana Schmitz[1], Victoria Lee[1], Xanthe L. Metcalf[1], Sean M Grimmond[3], Kym Pham Stewart[3], Mehreen Arshi[1], Angela M Steinmann[1], Nicola Blackburn[1], Ruth J. Lyons[1]

[1]The Kinghorn Cancer Centre, Garvan Institute of Medical Research, 370 Victoria Street, Darlinghurst, Sydney, New South Wales 2010, Australia.
[2]Royal North Shore Hospital, Westbourne Street, St Leonards, New South Wales 2065, Australia.
[3]University of Melbourne Centre for Cancer Research, Victorian Comprehensive Cancer Centre, 305 Grattan Street, Melbourne, Victoria, 3000, Australia
